# Cooperative adaptation to therapy (CAT) confers resistance in heterogeneous non-small cell lung cancer

**DOI:** 10.1371/journal.pcbi.1007278

**Published:** 2019-08-26

**Authors:** Morgan Craig, Kamran Kaveh, Alec Woosley, Andrew S. Brown, David Goldman, Elliot Eton, Ravindra M. Mehta, Andrew Dhawan, Kazuya Arai, M. Mamunur Rahman, Sidi Chen, Martin A. Nowak, Aaron Goldman

**Affiliations:** 1 Département de mathématiques et de statistique, Université de Montréal, Montréal, Canada; 2 Department of Physiology, McGill University, Montréal, Canada; 3 Program for Evolutionary Dynamics, Harvard University, Cambridge, Massachusetts, United States of America; 4 Computational Genomics Division, Arrayo, Boston, Massachusetts, United States of America; 5 7730E BlackCrest Pl., Tucson, Arizona, United States of America; 6 Division of Engineering in Medicine, Brigham and Women’s Hospital, Boston, Massachusetts, United States of America; 7 Interventional Pulmonology and Critical Care Medicine, Apollo Speciality Hospitals, Bengaluru, India; 8 Neurological Institute, Cleveland Clinic, Cleveland, Ohio, United States of America; 9 JSR Life Sciences Corporation, Tsukuba, Japan; 10 MBL International, Woburn, Massachusetts, United States of America; 11 Department of Genetics and Systems Biology Institute, Yale University, New Haven, Connecticut, United States of America; 12 Department of Medicine, Harvard Medical School, Massachusetts, United States of America; 13 Integrative Immuno-Oncology Center, Mitra Biotech. Woburn, Massachusetts, United States of America; Queen’s University Belfast, UNITED KINGDOM

## Abstract

Understanding intrinsic and acquired resistance is crucial to overcoming cancer chemotherapy failure. While it is well-established that intratumor, subclonal genetic and phenotypic heterogeneity significantly contribute to resistance, it is not fully understood how tumor sub-clones interact with each other to withstand therapy pressure. Here, we report a previously unrecognized behavior in heterogeneous tumors: cooperative adaptation to therapy (CAT), in which cancer cells induce co-resistant phenotypes in neighboring cancer cells when exposed to cancer therapy. Using a CRISPR/Cas9 toolkit we engineered phenotypically diverse non-small cell lung cancer (NSCLC) cells by conferring mutations in Dicer1, a type III cytoplasmic endoribonuclease involved in small non-coding RNA genesis. We monitored three-dimensional growth dynamics of fluorescently-labeled mutant and/or wild-type cells individually or in co-culture using a substrate-free NanoCulture system under unstimulated or drug pressure conditions. By integrating mathematical modeling with flow cytometry, we characterized the growth patterns of mono- and co-cultures using a mathematical model of intra- and interspecies competition. Leveraging the flow cytometry data, we estimated the model’s parameters to reveal that the combination of WT and mutants in co-cultures allowed for beneficial growth in previously drug sensitive cells despite drug pressure via induction of cell state transitions described by a cooperative game theoretic change in the fitness values. Finally, we used an *ex vivo* human tumor model that predicts clinical response through drug sensitivity analyses and determined that cellular and morphologic heterogeneity correlates to prognostic failure of multiple clinically-approved and off-label drugs in individual NSCLC patient samples. Together, these findings present a new paradox in drug resistance implicating non-genetic cooperation among tumor cells to thwart drug pressure, suggesting that profiling for druggable targets (i.e. mutations) alone may be insufficient to assign effective therapy.

## Author summary

Here, we provide mathematical and empirical evidence to support a potentially new paradigm in drug resistance, which we have termed “cooperative adaptation to therapy” (CAT). CAT is defined by a phenomenon wherein drug-sensitive cancer cells with different genetic and phenotypic features within a 3-dimensional heterogeneous tumor induce non-mutational resistance in their neighboring cells under pressure of cancer therapy. To develop this novel conclusion we deployed an interdisciplinary effort including an *ex vivo* human tumor model, a CRISPR/Cas9 platform with 3-dimensional *in vitro* experiments, and high throughput flow cytometry. Importantly, we wove these data together using a mathematical model of intra- and interspecies competition to understand how tumor heterogeneity influenced our observations. By estimating the model’s parameters, we determined that the combination of genetic clonal variants in co-cultures allowed for previously drug-sensitive cells to continue to grow despite drug pressure. We were thus able to characterize distinct growth regimens in mono- and co-cultures without and with drug pressure.

## Introduction

Lung cancer is the leading cause of cancer-related death in the United States, and non-small cell lung cancer (NSCLC) accounts for the majority of lung cancer cases each year [1]. Recent advances in the molecular understanding of NSCLC progression have informed the development of new targeted therapies that are safer and more effective than standard chemotherapy: of the nearly two-thirds of patients who have an oncogenic driver mutation, about half have of these are druggable [2]. ATP-competitive small molecule inhibitors of mutant epidermal growth factor receptor (e.g., erlotinib, gefinitib, afatinib, osimertinib), of mutant serine/threonine kinase b-raf (e.g., vemurafenib, dabrafenib), and of mutant anaplastic lymphoma kinase or of ROS1 proto-oncogene receptor tyrosine kinase (e.g., crizotinib, ceritinib, alectinib) have been approved for management of NSCLC [3]. Despite the increasing number of agents that have entered the clinic, successful treatment of NSCLC is hampered by drug resistance [4].

Two primary forms of resistance are predominantly studied: intrinsic and acquired [5]. Intrinsic resistance is largely considered to be due to aberrations including somatic mutations and DNA amplifications, which render primary therapy failures [6]. Acquired resistance, however, can emerge under selective pressures, and during treatment [7]. More recently, phenotypic plasticity and the role of ‘adaptive’ and drug-induced cell state transitions have introduced a new paradigm in acquired drug resistance [8, 9]. It is increasingly clear that an underlying driver of acquired resistance is due to the dramatic range of genetic and phenotypic diversity that is conferred during tumor evolution, comprising both passenger and driver mutations [10]. This heterogeneity within tumors, termed intratumor heterogeneity (ITH), can significantly impact responses to therapy and sensitivities to certain targeted agents, such as those listed above [11]. In NSCLC, intrinsically resistant tumor subpopulations can expand, or drug-tolerant cells can acquire alterations that confer more robust resistance to therapy [4, 12–15]. To improve the effectiveness of cancer therapeutics, it is therefore critical to understand whether, and to what extent, phenotypically distinct cells within the same tumor interact to promote resistance.

In combination with experimental approaches, quantitative methods arising from evolutionary theory have contributed to our understanding of the role of genetic heterogeneity in tumor initiation, progression, and resistance [16–24]. Mathematical models have previously demonstrated that local cell density and cell cycle can contribute to spatiotemporal heterogeneity and differences in cell response to treatment [25]. However, applying computational approaches to examine phenotypic heterogeneity has been less well pursued [26]. Here, we explore how intratumor phenotypic heterogeneity in a model of NSCLC affects the acquisition of resistance and population growth. To generate phenotypically unique sub-clones, we use the CRISPR/Cas9 system to stochastically mutate Dicer1, a cytoplasmic riboendonuclease responsible for maturation of microRNA [27]. Indeed, dysregulation of microRNA is heavily implicated in NSCLC [28]. Using a three-dimensional *in vitro* culture platform, which most closely parallels *in vivo* growth, we show that intrinsically-sensitive, phenotypically distinct NSCLC sub-clones support each other to rapidly evolve a therapy-resistant phenotype via drug-induced cell state transitions, a behavior we term “cooperative adaptation to therapy” (CAT). We develop an *in vitro*-validated model of intra- and interspecies competition governed by replicator dynamics [29] to simulate how CAT affects population growth over time. These findings build on previous evidence that drug-induced phenotypic transitions can underpin resistance, and implicate a population-wide impact of this phenomenon.

## Methods

### Ethics statement

Anonymous non-small cell lung cancer (NSCLC) tissue samples were collected under IRB approval with due written consent from each patient.

### Chemicals and reagents

Unless noted otherwise, all reagents, small molecule inhibitors and chemotherapies were of the highest grade purchased from Sigma-Aldrich (St. Louis, MO). The NCI Diversity Set VI was used to screen cells against clinically approved drugs for cancer therapy [30]. All chemotherapeutics and small molecule inhibitors were dissolved in DMSO to a stock concentration of 10mM and kept frozen.

### Cell culture

Parental cell lines were generated as previously described [31] as a clone LCC1.11, with KrasG12D;p53-/-;Dicer1f/+, where Dicer 1 is heterozygous. Dicer1 mutant clones were generated by transfecting the parental clone with CRISPR plasmid targeting the Dicer1 locus, and selection and expansion of single colonies. Lentiviral particles expressing codon optimized fluorescent proteins under suCMV promoter were transfected into cell lines following manufacturer protocol (GenTarget, San Diego, CA). A blasticidin gene under RSV promoter was used to select positively transduced cells. Despite multiple rounds of selection, there were some noticeable cells ‘negative’ for the fluorescent protein expression, or populations of cells that remained at a low confident level of expression, which were subsequently excluded from analysis in flow cytometry (see flow cytometry section, below).

### Human explant studies

Anonymous human NSCLC tissues were assessed by CANscript using fresh specimen. Fresh tumor tissues were collected immediately after surgical resection. The tumor samples were transported to the laboratory at 4°C, in appropriate transport buffer within 60 minutes post-resection, for *ex vivo* studies and molecular and pathological evaluations. Tissues were cut into thin sections and cultured in 48-well plate using optimized conditions. Tumors were treated with the indicated drugs at the clinical max concentration (Cmax) for 72 hours. DMSO was used as a vehicle control. Tissue was then formalin fixed and paraffin embedded (FFPE) for subsequent analyses.

Quantifying intratumor phenotypic, morphologic, and cellular heterogeneity was performed by visual inspection of a clinical pathologist (Dr. David Goldman, MD, co-author on the present study) using the following methodology: FFPE tissue sections were stained with hematoxylin and eosin (H&E) to identify the respective nuclear DNA content and cytoplasm of cells in the tissue. 1) In an effort to quantify the outgrowth of different tumor ‘clones’, a clinical pathologist then counted the number of histologically distinct tumor ‘neighborhoods’, which were defined as grossly-distinct (clusters of tumor cells growing within a confined region and sharing unique distinguishing morphology from other clusters of tumor cells in the same visual field). 2) Within each tumor ‘neighborhood’, cells were scored based on nuclear density and uniformity as well as cellular morphology uniformity on a scale of 1-5, where 5 is the most distinct and 1 is the most similar. 3) A score for cellular and morphologic heterogeneity was developed by adding together the value attributed to each ‘neighborhood’ for nuclear content uniformity and cellular morphology uniformity (2), which was multiplied by the number of respective tumor neighborhoods (1) for that tissue.

Predicting response to therapy was performed using a clinically trained algorithm that was previously described [32]. Briefly, multiple terminal and kinetic assay (tumor morphology, tumor cell proliferation, cell death, viability, cell growth, and metabolic status) inputs were trained in a proprietary machine learning algorithm [32]. The algorithm generates a single score (currently defined as M-Score, but previously published under the nomenclature S-Score) for each drug arm tested. An M-Score > 25 indicates positive response and a value of M-Score ≤ 25 is indicative of a negative response.

### Flow cytometry analyses

Cells were cultured as indicated above using the tumor spheroid NanoCulture plates (MBLI, Woburn MA). To assess the total number of cells growing in the 3-D spheroid versus aberrant 2-D growth, cells were plated and counted every other day for 7 days. Cells were imaged by brightfield microscope and 2-D adherent were quantified. Number of 2-D growing cells were quantified as % of total cells growing in culture. Less than 2% of cells on any day were noted to grow in 2-D versus the majority (98%-99% of cells) growing in the 3-D tumor spheroids. Cells were cultured in various proportions with either the parent WT cell line or any one of a combination of the different Dicer1 mutants. Cells were removed from culture dishes with StemPro Accutase dissociation reagent (Invitrogen, Carlsbad CA) and fixed with 4% paraformaldehyde in PBS for 30 minutes at RT. Cells were then processed using the BD Fortessa 4 laser, 17 parameter flow cytometer. Measurements were made in the indicated fluorescent channel based on the fluorescent tag (typically blue fluorescent protein (BFP) and red fluorescent protein (RFP)), and any overlap was compensated prior to analysis. A gating strategy was employed to select cells based on the side scatter (SSC) and forward scatter (FSC) from the vehicle-treated parental population, followed by a selection of singlets based on FSC width and height. Propidium iodide exclusion was used to validate viable cells are contained within the FSC:SSC gate. An equal volume of cells was analyzed for each experiment and events were recorded in the defined gates (described above) and in the correct fluorescent channels. Data analysis was performed using FlowJo software (Tree Star Inc., Ashland OR). Experiments were performed a minimum of three times (biological replicates) on independent days. Notably, and despite blasticidin selection, there were identifiable populations of cells that expressed no detectable fluorescent tag. This was attributed to heterogeneity of both CMV infection, expression and blasticidin selection. During analysis in flow cytometry, there was no increase, decrease or change in the proportion of labeled to unlabeled cells in the vehicle treatment or drug treatment cohorts.

### RNA sequencing

RNA was isolated from cells that were cultured in 3-D culture after 48 hours of growth in spheroids. Cells were rinsed and then lysed followed by RNA extraction using manufacturer protocol (Qiagen, Hiden Germany).

### Library preparation and sequencing

RNA libraries were prepared using Illumina TruSeq Stranded mRNA sample preparation kits from 500ng of purified total RNA according to the manufacturer’s protocol. The resultant dsDNA libraries were quantified by Qubit fluorometer, Agilent TapeStation 2200, and RT-qPCR using the Kapa Biosystems library quantification kit according to manufacturer’s protocols. Uniquely indexed libraries were pooled in equimolar ratios and sequenced on a single Illumina NextSeq500 run with single-end 75bp reads by the Dana-Farber Cancer Institute Molecular Biology Core Facilities.

### RNAseq analysis

Sequenced reads were aligned to the UCSC mm9 reference genome assembly and gene counts were quantified using STAR (v2.5.1b) [33]. Differential gene expression testing was performed by DESeq2 (v1.10.1) [34] and normalized read counts (FPKM) were calculated using cufflinks [35]. RNASeq data was mapped to a reference obtained from NCBI GRCh38.p12. Indexing of the raw sequence data contained in GRCH38.p12 was performed by the STAR open source alignment tool. These services were run on the Amazon Web Service (AWS) Elastic Cloud Compute (EC2) infrastructure with 8 vCPU cores and 32 GB Ram. Mapped results were correlated to index files using STAR 2pass. Indels were realigned and bases recalibrated before variant calling by STAR. We utilized the HaplotypeCaller as outlined in GATK best practices with the Java implementation. Variant calls (.vcf) were generated and annotated back to the GRCh38.p12 genomic reference assembly in order to determine point mutations.

### Modeling the population dynamics of adaptive resistance in mono- and co-cultures

The mathematical model of adaptive resistance consists of two ordinary differential equations describing intra- and inter-species phenotypic switching founded upon game theoretic interaction assumptions identical to the Lotka-Volterra model commonly used in ecology [36, 37]. This choice is founded upon well-developed mathematical modelling approaches to understanding, for example, the dynamics of interacting microbes and cancer cell populations [38, 39]. Full details of the model are provided in the Supplementary Information file S1 SuppInfo. Briefly, we consider a wild *x*_WT_ and mutant $x_{{\text{M}}_{i}}$ (*i* = 1, 2, 3) type. Let *b* be the birth rate of cells and *d* be their rate of death. In absence of drugs, cells initially grow exponentially at rate *l*_*type*_ (*type* = *WT*, *M*_*i*_), where *l*_*type*_ is the effective birthrate, or fitness, given by *l*_*type*_ = *b* − *d*. Cell numbers eventually saturate at *K*_*type*_ (the population’s effective carrying capacity, where $K=\tilde{K}\left(1-d/b\right)$ with $\tilde{K}$ being the standard carrying capacity–see S1 SuppInfo for extended details). Based on the observation of a temporary decline and eventual rebound in the monoculture growth assays after drug pressure was applied, we assumed each cell type is capable of developing an intra-species drug tolerant phenotype under drug pressure [40, 41] that we termed ‘intra-species phenotypic switching’. The intra-species growth dynamics can then be summarized by

$$\begin{array}{c} \begin{array}{cc} \frac{dx_{type}^{sens}}{dt} & =l_{type}^{sens}x_{type}^{sens}(1-\frac{\left(x_{type}^{sens}+x_{type}^{tol}\right)}{K_{type}})-\nu_{type}x_{type}^{sens}\\ \frac{dx_{type}^{tol}}{dt} & =l_{type}^{tol}x_{type}^{tol}(1-\frac{\left(x_{type}^{sens}+x_{type}^{tol}\right)}{K_{type}})+\nu_{type}x_{type}^{sens}, \end{array} \end{array}$$ (1)

where the superscript *sens* describes the drug-sensitive phenotype, and *tol* the drug-tolerant phenotype; *ν*_*type*_ measures the intra-species switching rate. Note that no backwards switching from tolerant to sensitive is considered.

We assume that intra-species interactions are dominated by inter-species relationships, that is to say that the adaptive behaviors of cells in co-culture are dictated by CAT dynamics. Co-culture dynamics were modelled as

$$\begin{array}{c} \begin{array}{cc} \frac{dx_{\text{WT}}}{dt} & =l_{1}x_{\text{WT}}(1-\frac{x_{\text{WT}}}{K_{WT_{co}}}+a_{12}x_{{\text{M}}_{i}})\\ \frac{dx_{{\text{M}}_{i}}}{dt} & =l_{2}x_{{\text{M}}_{i}}(1-\frac{x_{{\text{M}}_{i}}}{K_{Mi_{co}}}+a_{21}x_{\text{WT}}), \end{array} \end{array}$$ (2)

where $x_{\text{WT}}=x_{\text{WT}}^{sens}+x_{\text{WT}}^{tol}$, $x_{{\text{M}}_{i}}=x_{{\text{M}}_{i}}^{sens}+x_{{\text{M}}_{i}}^{tol}$, $K_{WT_{co}}$ and $K_{Mi_{co}}$ are the carrying capacities of the WT and mutant in co-culture, and the terms *a*_12_ and *a*_21_ denote the interactions between cell types.

In summary, we assumed the following:

- In absence of drugs, monoculture growth is initially exponential and eventually saturates at an effective carrying capacity specific to each cell type (logistic growth). Mathematically, we assume that $l_{type}^{sens},K_{type}>0$ and all other parameters in (Eq 1) are equal to 0.
- Under exposure to drugs, sensitive cells in monoculture can perform intra-species switches to drug tolerant type at rate *ν*_*type*_. The effective carrying capacity *K*_*type*_ remains unchanged from the non-drug case, however $l_{type}^{tol},\nu_{type}$ are no longer assumed to be identically equal to zero, and $l_{type}^{sens}$ may be negative to account for decreased fitness in the presence of the drug.
- In the presence of the therapeutic stresses, sensitive subtypes phenotypically switch into more drug tolerant/resistant types. Further, each phenotypic trait is heritable, that is that daughter cells have the same phenotype.
- Intra-species growth dynamics are weaker than inter-species co-operative adaptation to therapy, so that the inter-species interaction terms *a*_12_ and *a*_21_ dominate. This implies that the additional co-culture effects are not changing or modifying the intra-species phenotypic switching mechanism but rather inducing a game theoretic change in the fitness values due to genotype-genotype interactions. Mathematically, we hypothesized that, as *x*_WT_ and $x_{{\text{M}}_{i}}$ denote the total number of tolerant and sensitive cells, intra-species transformation events are inconsequential based on the dynamics defined by (Eq 2).
- The proliferation potentials and apoptosis rates of the sensitive and drug-tolerant subtypes are dose-dependent (note, however, that since only a single dose was considered experimentally, we fixed the dose and did not elaborate the dose-response function of each genotype).

### Adaptive resistance model parameter optimization

The mathematical model’s parameter values were estimated from the experimental procedures described in S1 SuppInfo. To perform the optimization, we used the Matlab function *fmincon* that minimizes the objective (cost) function of a nonlinear model with constraints [42]. To reduce the influence of initial conditions on the outcome, goodness-of-fit was assessed by minimizing over the resulting set of cost function values obtained via a multi-step parameter estimation procedure, as described in the Supplementary Information file S1 SuppInfo.

## Results

### Establishing a model of phenotypic heterogeneity, *in vitro*

**Fig 1:**
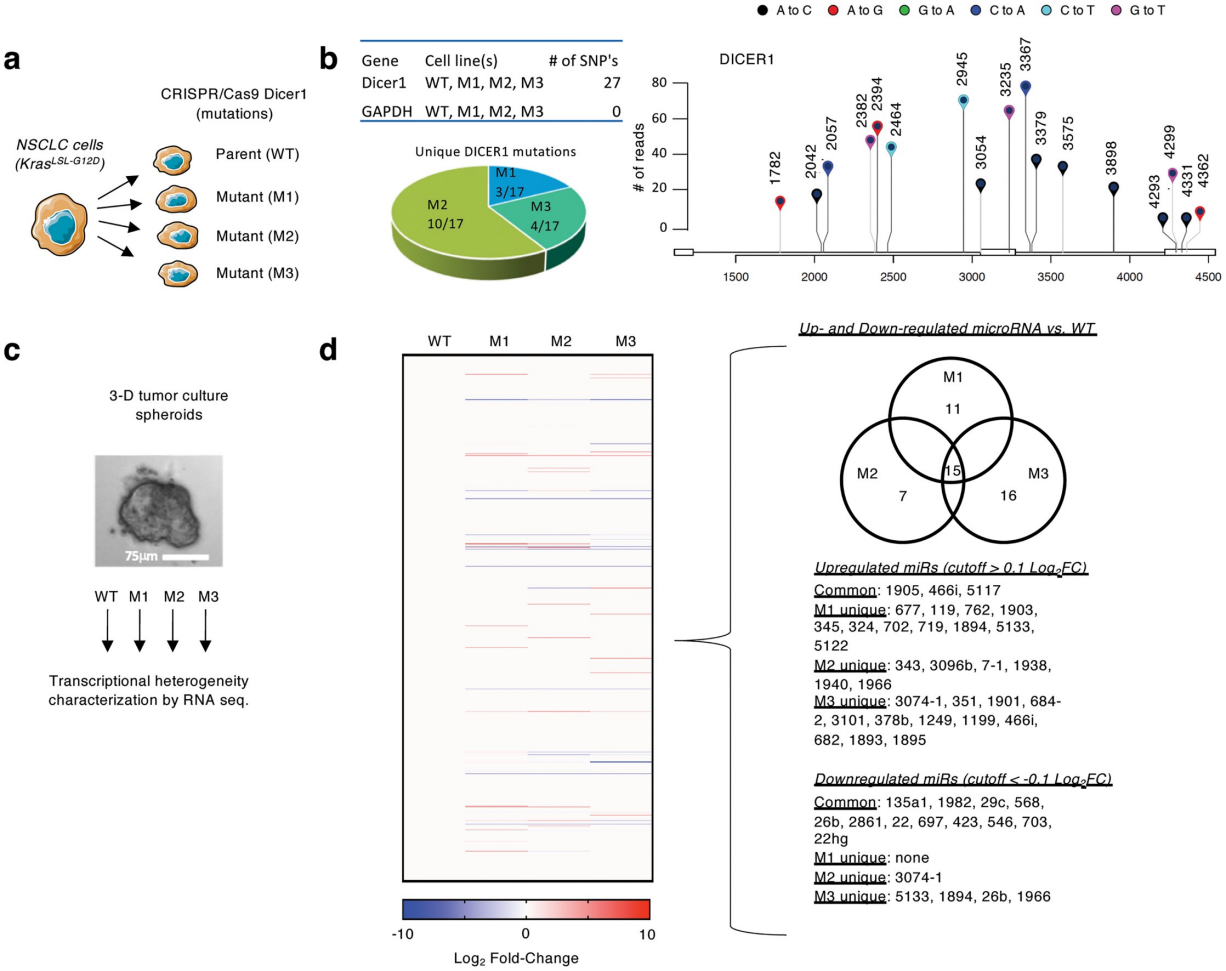
Characterizing phenotypic heterogeneity in Dicer1 engineered NSCLC cells. **a**) Murine-derived non-small cell lung cancer (NSCLC) cells were engineered with mutant Dicer1 clones using the CRISPR/Cas9 toolkit. **b**) Lollipop graph quantifies SNPs in the mutant vs. WT cells including location and mutation annotation. Total number of normalized SNP counts is shown on the Y-axis. Table shows number of SNP identified in each cell line, GAPDH was used as a control to validate SNP identification algorithm. **c**) 3D cultured cells were characterized for transcriptional and therapeutic sensitivity heterogeneity. **d**) (left) Heat map shows differential expression pattern of miRNA compared to WT; (right) List of unique and shared differentially expressed miRNA.

To study the role of intratumor heterogeneity in therapy failure, we first derived and cloned a cell line [43]. This cell line possesses an oncogenic Kras in conjunction with homozygous p53 and heterozygous Dicer1 loss of function (Kras G12D/+;p53 / ;Dicer1 +/) and is capable of inducing tumors when transplanted into immunocompromised mice. Because Dicer1 regulates the production of miRNA that can dramatically influence the phenotypic heterogeneity of a cell [44, 45], we engineered clones using the CRISPR/Cas9 toolkit to introduce a variety of mutations in Dicer1 (Fig 1a). Confirmation of Dicer1 sequence mutations were determined using NCBI reference data, identifying insertions and deletions as well as 17 unique single nucleotide polymorphisms (SNPs) in the mutant cell lines compared to WT (Fig 1b and S1 Fig).

The mutants and WT cell lines were cultured in a physiologically-relevant manner *in vitro*, using a three-dimensional nano-culture platform that provided a substrate-free growth surface containing uniform, nanofabricated imperfections, which forced spheroid growth (NanoCulture plates –Fig 1c). NanoCulture plates enabled better long term growth potential than ultra low adherence (ULA) plates, and growth of spheroids were also more uniform (S2 Fig). Using this approach, tumor spheroid growth is not confounded by external factors such as matrigel or collagen.

We characterized transcriptional heterogeneity of the wild type (WT) and mutant (M1-3) cells via interrogation of the mRNA sequencing from 3D cell cultures (Fig 1c). Primarily, differential expression analysis confirmed Dicer1 target-gene expressions (i.e. miRNA) were significantly, statistically different among mutants compared to WT with varying expression profiles (Fig 1d). Differential expression profiling of the top and bottom 500 dysregulated genes in each cell line confirmed unique transcriptional heterogeneity was observed in each mutant cell line with many reaching statistical significance (p-adjusted < 0.05) (see S1 Table).

### Experimentally-observed multi-drug resistance in Dicer1 wild-type and mutant heterotypic co-cultures

**Fig 2:**
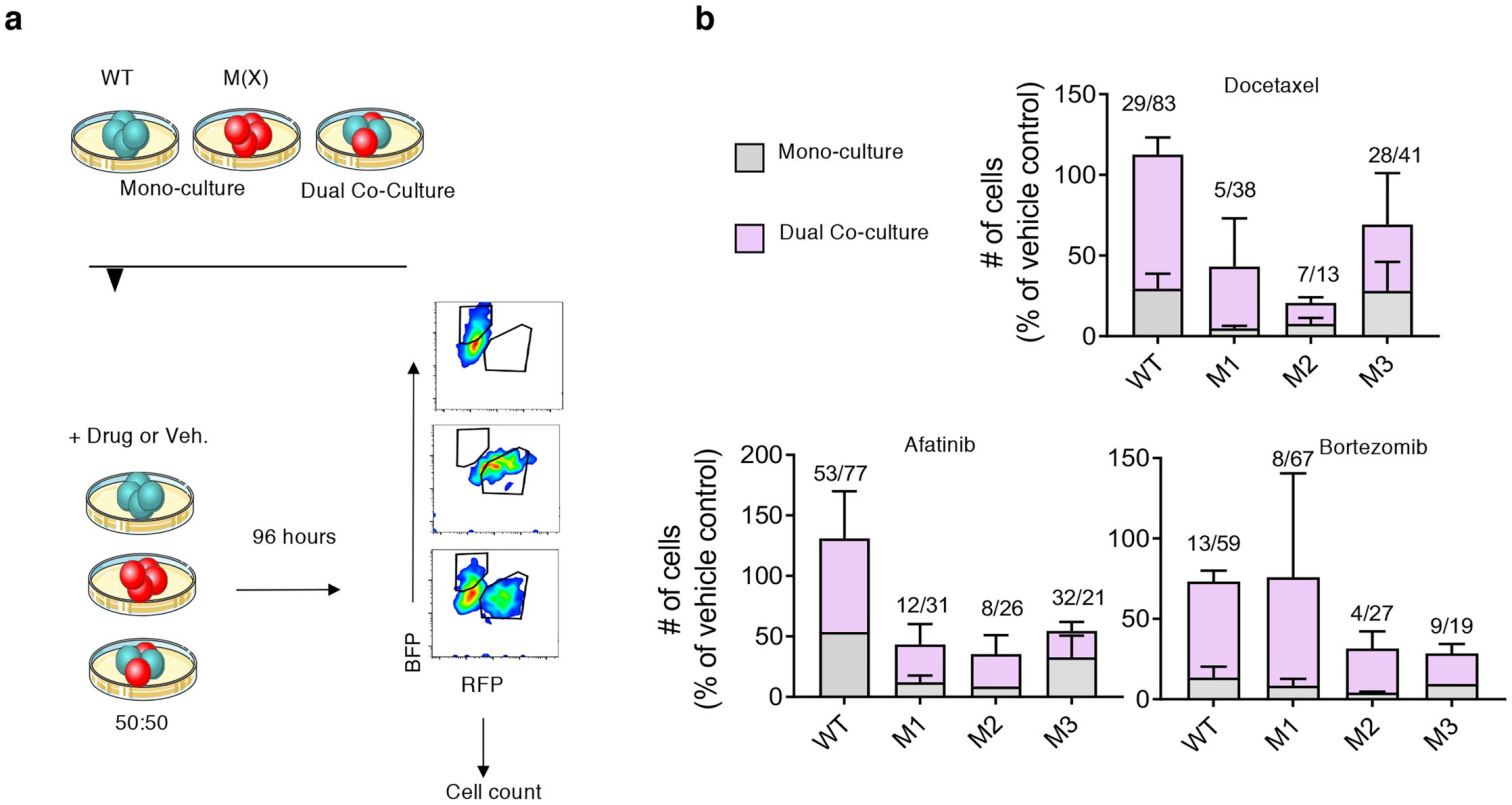
*In vitro* tumor spheroid growth of wild-type and Dicer1 mutant NSCLC clones. a) Experimental design schematic. Fluorescently-tagged WT or mutant NSCLC clones (M1, M2 or M3), designated as M(X), were cultured alone or in a heterotypic 3-D co-culture (WT+M) at 50:50. Drugs or vehicle control were introduced to culture for 96 hours followed by flow cytometry and count of gated cells (see Methods for details). b) Stacked histograms quantify the number of cells in each clone remaining in spheroids after 96 hours drug treatment in mono-culture or mixed, dual co-culture with WT. WT stacked bar graph indicates mono-culture or co-culture with mutants. Values above columns indicate the % mean in monoculture/co-culture. Experiments performed in biological replicate on independent occasions (N>3 in each group). Error bars indicate standard error from the mean.

In order to study how clonal diversity might impact response to drugs (sensitivity or resistance) we grew Dicer1 mutant clones and parental wild-type in separate mono-cultures or mixed co-cultures in 3-D and exposed them to different drugs at lethal concentrations. Using lentiviral-transfected fluorescent ‘tags’, flow cytometry was deployed to quantify the abundance of the different mutant clones in dual co-cultures, as described in the methods section (Fig 2a). We cultured the pre-labeled clones alone or together with WT in 3-D culture at a ratio of 50:50 in the presence or absence of multiple clinically-relevant anticancer drugs: docetaxel, bortezomib, and afatinib [46]. Primarily, we determined that, while all cell lines showed heterogeneous sensitivities to drugs in mono-cultures as indicated by the proportion of tumor cells in spheroids after 96 hours of drug exposure, a mutant-WT mixed co-culture resulted in an increased proportion of each clone relative to the vehicle control (Fig 2b). The data indicated that both cell lines ‘benefitted’ in a mixed co-culture (WT or mutant), increasing their relative ‘resistance’ to drug compared to the same cell line’s mono-culture. This finding was consistent in each drug tested and in every mixed culture, with the exception of the M3/WT co-culture exposed to afatinib.

### Model development to study clonal growth dynamics

**Fig 3:**
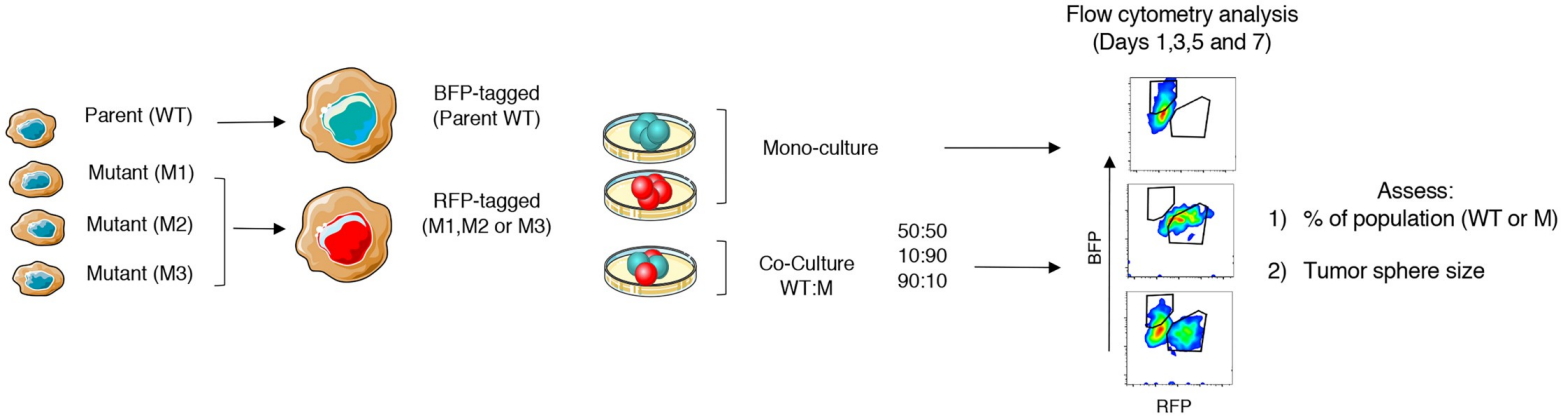
Lentiviral tagging and flow cytometry strategy. WT and mutant cells were induced to constitutively express blue and red, respectively, fluorescence by lentiviral transfection. Cells were then cultured alone (monocultures) or WT and mutant together (co-cultures) in 3-D culture at density ratios of 50:50, 90:10, and 10:90. The % of cells in the tumor sphere population and the size of the tumor sphere were measured by flow cytometry twice at 48 hour intervals in the presence or absence of drug.

Next, we sought a method to study the growth dynamics of the phenotypically diverse mutant clones in mono- and co-culture with parental WT cells in a competitive growth assay. We cultured the pre-labeled clones alone or together with WT in 3-D culture at different population density ratios of 50:50, 90:10, or 10:90. Using flow cytometry, we then performed two measurements at 48 hour intervals over the course of 7 days: 1) the % and number of cells in the tumor sphere population that is either WT or Mu, and 2) the size of the tumor sphere based on the number of fluorescently-detected, gated cells (Fig 3), which is indicative of overall tumor sphere growth. These data were integrated into the subsequent math modeling experiments and computational analyses described in the following sections.

### Genotype interactions induce a cooperative adaptation to therapy (CAT)

**Fig 4:**
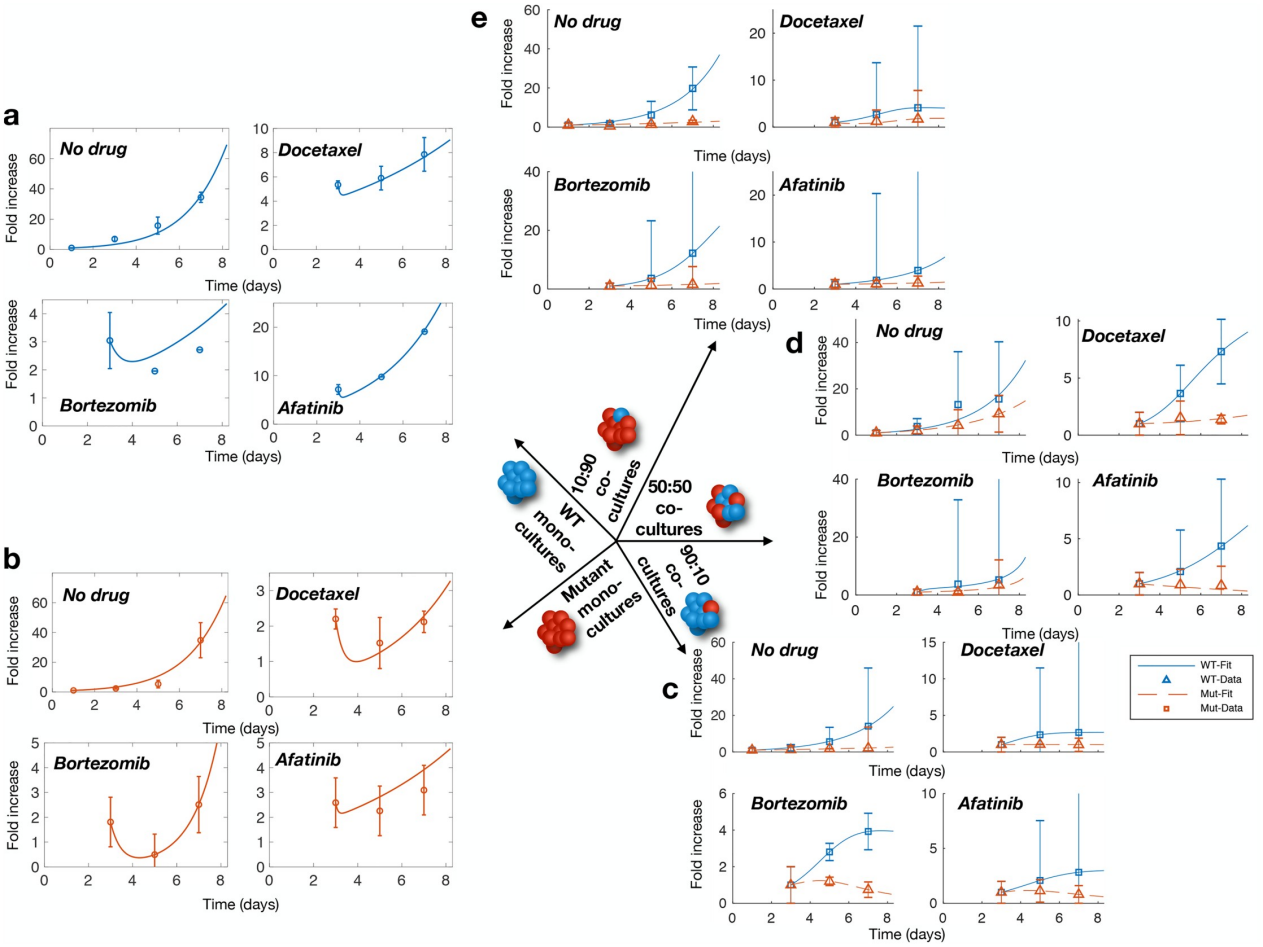
Population dynamics in mono- and co-cultures. **a)** WT monocultures growth without drug pressure, in docetaxel, in afatinib, and bortezomib (from top left to bottom left). **b)** M1 monoculture growth without drug pressure, in docetaxel, in afatinib, and bortezomib (from top left to bottom left). **c)** M1 90:10 growth without drug pressure, in docetaxel, in afatinib, and bortezomib (from top left to bottom left). **d)** M1 50:50 growth without drug pressure, in docetaxel, in afatinib, and bortezomib (from top left to bottom left). **e)** M1 10:90 growth without drug pressure, in docetaxel, in afatinib, and bortezomib (from top left to bottom left). Error bars represent normalized standard deviation of experimental data.

We leveraged the mathematical model to better understand the growth dynamics in the *in vitro* competitive growth assays. We first characterized monoculture dynamics (with and without drug) for the WT and all three mutants. In the absence of drug pressure, cells initially grew exponentially in monoculture (Fig 4a and 4b, top left) with no suggestion of intra-species phenotypic switching (“No Drug” columns in Table 1). However, the ‘dip and rebound’ observed in the monoculture growth assays after the addition of docetaxel, bortezomib, or afatinib was successfully captured through the monoculture model in (Eq 1) (Fig 4a and 4b top right, bottom; Drug columns in Table 1—see S1 SuppInfo). Our estimates predicted decreased growth of the sensitive phenotype and increased growth of the tolerant subtype in the presence of drugs in monoculture. The complete results of the monoculture parameter estimation procedure are reported in Table 1.

**Table 1:** Monocultures without and with drugs.

	No Drug				Docetaxel				Bortezomib				Afatinib			
Parameter	WT	M1	M2	M3	WT	M1	M2	M3	WT	M1	M2	M3	WT	M1	M2	M3
$l_{type}^{sens}$	0.5988	0.6499	0.714	0.6676	-2.776	-1.643	-0.8176	–	-3.6713	-1.9917	-1.9874	-1.9807	-1.9948	-1.9921	-1.9984	-1.7984
*K*_*type*_	999	999	999	999	999	999	999	–	999	999	999	999	999	999	999	999
$l_{type}^{tol}$	0	0	0	0	0.144	0.1581	0.001	–	0.3404	0.0233	-0.7449	-1.9807	0.1644	0.8394	-0.0458	0.1091
*ν*_*type*_	0	0	0	0	9.7352	0.8641	9.9977	–	9.5633	0.5724	0.5724	7.837	1.8546	0.1441	2.5399	9.9975
Error	1E-3	1E-3	1E-3	1E-3	1E-3	1E-3	1E-3	–	1E-3	1E-3	1E-3	1E-3	1E-3	1E-3	1E-3	1E-3

As previously described, we hypothesized that genotype-genotype interactions are the dominant growth mechanism in co-culture (see Eq (2)). This assumption was borne out as the model successfully captures the WT and mutant growth dynamics in co-culture mixes (10:90, 50:50, 90:10 WT:Mu), as shown for M1 in Fig 4, and for fixed proportion (50:50) across all mutant co-culture mixes (S2 Table and S4 Fig), with the exception of the previously identified M3/WT 50:50 co-culture exposed to afatinib.

**Fig 5:**
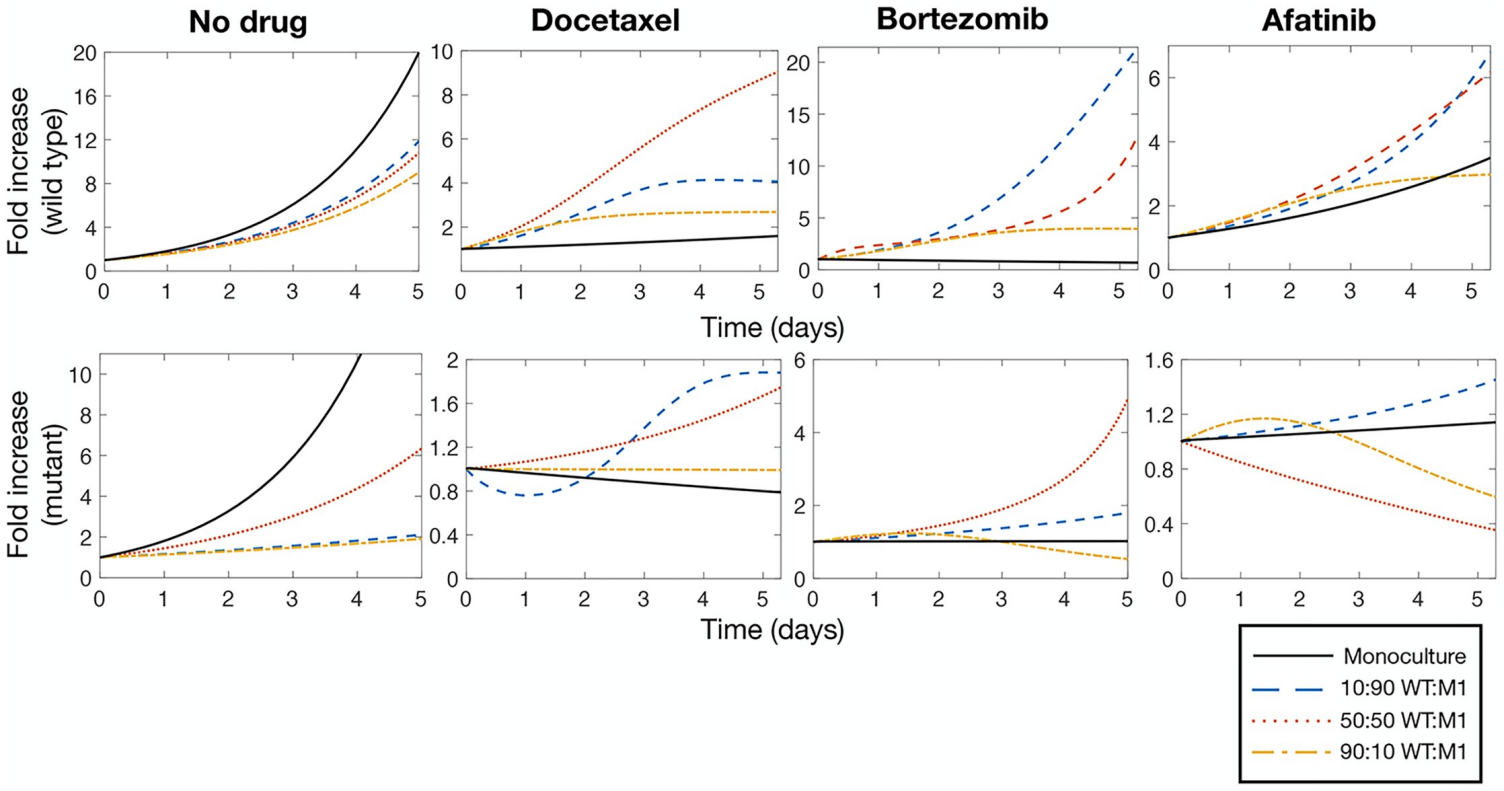
Heterogeneity is beneficial to growth during drug exposure. In absence of drug pressure, monoculture growth (solid black line) for both WT and M1 is significantly faster than all three co-cultures of 10:90 (dashed blue line), 50:50 (dotted red line), and 90:10 (dashed-dotted yellow line) mixes of WT:M1. However, when exposed to docetaxel, bortezomib, and afatanib, co-culture heterogeneity is beneficial to both cell types, conferring faster growth than in the monoculture case. Top: model predictions of WT growth; bottom: model predictions of M1 growth.

Next, we tested the hypothesis that cancer cells induce rapid adaptations within and among neighboring cells to improve fitness of the heterogeneous population under drug pressure and thwart destruction. That is to say, cancer cells behave in a ‘cooperative’ manner to promote drug resistance in neighboring cells that do not have similar genetic and phenotypic features, hereafter referred to as cooperative adaptation to therapy (CAT). We hypothesized that the form of ‘adaptive’ resistance describing monoculture growth does not impact on the overall co-culture dynamics (i.e. the additional co-culture effects induce a change in the fitness values due to genotype-genotype interactions in a cooperative game) [47]. Therefore, we leveraged the parameterized mathematical model for comparative analysis. For the four drug scenarios (no drug, docetaxel, bortezomib, and afatanib), we compared monoculture growth dynamics to those in all three co-culture proportions by simulating all scenarios with identical initial condition (*x*_WT_(0) = 1, $x_{{\text{M}}_{i}}\left(0\right)=1$) for the WT and M1 mutant. In the absence of drug exposure, for both cell types, we found that growth in monoculture is demonstrably stronger than in co-culture (Fig 5 left, top and bottom). However, under drug pressure, growth in co-culture quickly outpaces that of the monoculture (Fig 5 docetaxel, bortezomib, and afatanib panels, top and bottom). These results demonstrate mathematically that our model, parameterized to our *in vitro* NanoCulture growth assays, predicts that an advantageous drug tolerant phenotype, due only to heterogeneity, is conferred in co-culture. Indeed, these findings are consistent with the CAT hypothesis.

### Intratumor phenotypic heterogeneity correlates to multi-drug resistance in individual patients, *ex vivo*

**Fig 6:**
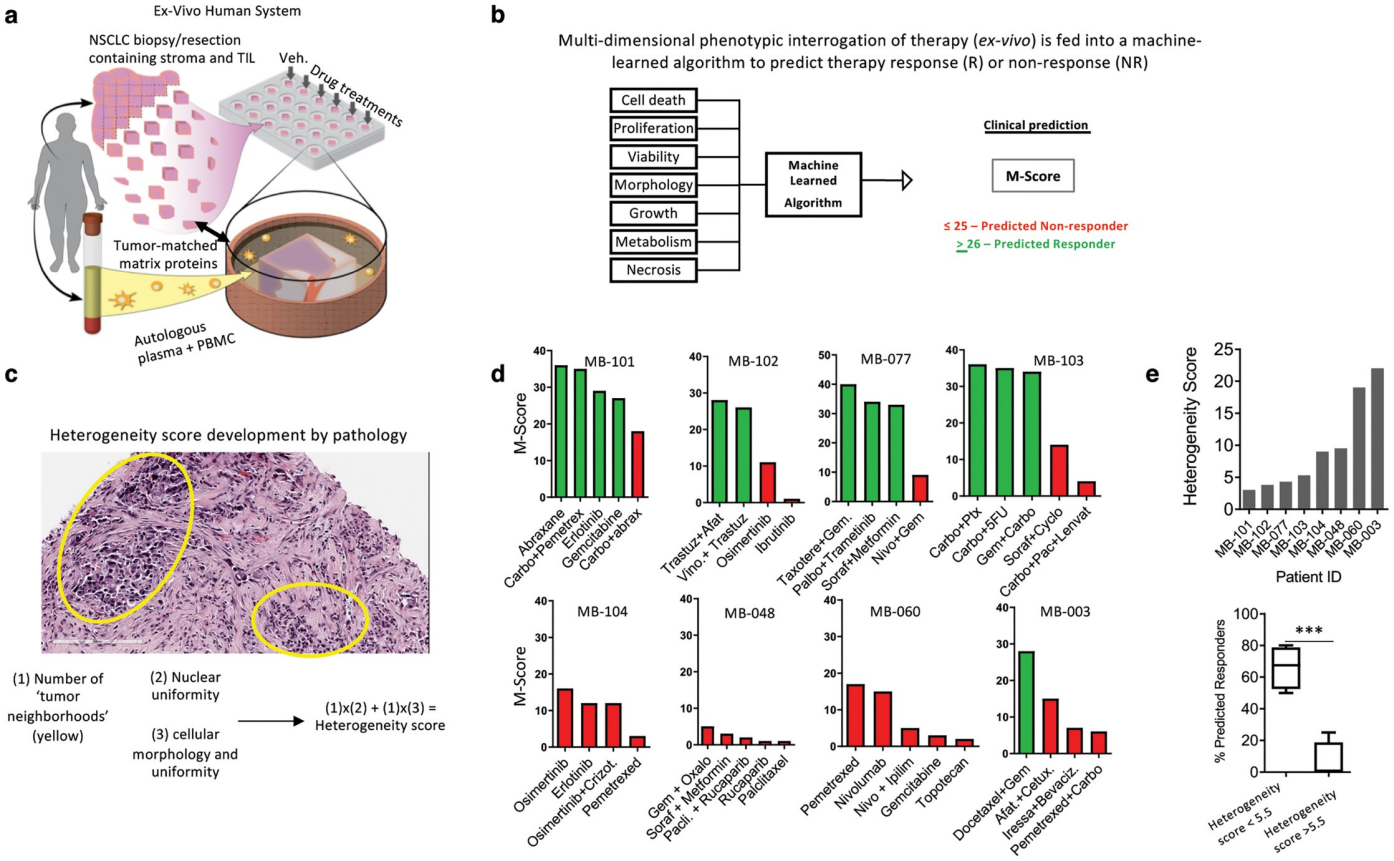
Assessing and quantifying ITH using CANscript. **a**) Tumor explants were generated from tumor biopsy isolated from NSCLC patients and were cultured *ex vivo* on matched tumor matrix proteins supplemented with autologous patient plasma. Illustration by Wendy Chadbourne, 2018 Inky Mouse Studios, www.inkymousestudios.com, provided under CC BY 4.0. **b**) clinical response prediction was performed using the M-Score; **c)** description of the development of a histology-based ‘heterogeneity score’ developed by pathology. Scores from 1-5 were collected by multiplying the number of histologically distinct tumor regions with cellular morphology and nuclear uniformity; **d)** stratified patient samples across a spectrum of heterogeneity scores and their predicted response to anticancer therapy based on the M-Score; **e)** histogram shows the heterogeneity score of all patient samples (N = 8). Lower panel shows box and whisker plot quantifying the % of predicted responder from clusters of patient samples with a heterogeneity score <5.5 (N = 4) or >5.5 (N = 4). ***p<0.001 by T-test.

The data produced to this point indicate that heterogenous tumors can overcome the onslaught of anti-cancer drugs by rapidly inducing resistance through co-supportive effects (i.e. CAT). We tested the hypothesis that ITH directly correlates to therapy response of many drugs, not just those predisposed by mutations. Indeed, ITH is a consequence of genetic and phenotypic (i.e. mutations giving rise to unique cellular and morphologic characteristics) variability among tumor cells, which is tightly associated with treatment resistance [48]. To do this, we deployed a human tumor explant platform that replicates the native tumor microenvironment including stromal and immune cells and allows for testing multiple drug responses in a single tumor explant [32]. First, tumor explants were generated from fresh biopsies isolated from NSCLC patients, cultured *ex vivo* on matched tumor matrix proteins supplemented with autologous patient plasma, and exposed to various clinically-relevant drug regimens (Fig 6a and S3 Table). This platform uses a trained algorithm that incorporates phenotypic response assays, which enables clinical response prediction (given by the M-Score) with published clinical accuracy (Fig 6b) [32].

Next, we wanted to quantify phenotypic heterogeneity by assessing the morphologic and cellular diversity of tumor cells using an assessment of clinical pathology. i.e. phenotypic rather than genetic heterogeneity. To do this, we developed a ‘heterogeneity score’ for each patient tumor sample based on the cellular and morphologic diversity of the tumor as quantified by a clinical pathologist using the following approach: Fresh tumor biopsies were stained with hematoxylin and eosin to visualize and differentiate the nucleus and cytoplasm of each cell. Next, a clinical pathologist (see Methods) quantified the number of distinct tumor ‘neighborhoods’, which are defined by clusters of tumor cells growing with similar morphologies within a single visual field at 20X magnification. This was performed on three independent tumor fragments from the same patient. Within each ‘neighborhood’ we then quantified, on a scale of 1-5, the uniformity of cell morphology and nuclear content in each visual field wherein 1 is more uniform and 5 is highly diverse. We then calculated the ‘heterogeneity score’ by multiplying the number of histologically distinct tumor neighborhoods from a single field-of-view with cellular morphology and nuclear uniformity, each on a scale of 1-5 (Fig 6c). The resulting score was used to stratify NSCLC patient samples across a spectrum of heterogeneity from low to high (Fig 6d). Interestingly, and consistent with evidence that ITH associates with therapy failure [48], we determined that the degree of ITH (as defined by our ‘heterogeneity score’) significantly affects predicted antitumor effect of multiple anticancer therapeutic regimens and combinations in an individual patient sample, as determined by the M-Score (Fig 6e, *p* < 0.001).

While this finding is compatible with previous evidence that heterogeneity across a population of patients associates to therapy failure [49, 50], these data provide evidence that in an *ex vivo* setting, within a single patient, numerous conventional treatment options are similarly ineffective when ITH is high, supporting the hypothesis that ITH and CAT are potentially linked.

## Discussion

Identifying the biomarkers and patterns that result in drug resistance is penultimate to eradicating therapy failure in cancer. To achieve this and to advance precision medicine, mutational profiling for ‘druggable’ targets has been an active area of investigation for oncology in the past several decades [51]. However, there is mounting evidence that non-mutational mechanisms result in drug resistance, regardless of the mutational load in tumors [52]. Indeed, we have outlined here a potentially new paradigm in drug resistance by showing how “cooperative adaptation to therapy” (CAT), due to ITH, can induce a drug tolerant phenotype, which has significant consequences for therapeutic response. We took an approach that combined experimental evidence with theoretical modeling, and constructed a replicator dynamics model of intra- and interspecies competition that can potentially explain how individual clones within a tumor lead to treatment resistance. We determined that the emergence of CAT is directly related to the presence of neighboring clonal subsets within a tumor that force de-novo drug resistance after drug exposure.

We investigated how phenotypic switching can occur within heterogenous tumors by leveraging a three-dimensional nanoculture *in vitro* spherical growth platform and Dicer1 mutants derived from CRISPR/Cas9 gene editing. We characterized tumor spheroid growth over 7 days in monocultures and co-cultures (in proportions of 10:90, 50:50, and 90:10 WT:Mutant) in the absence of drugs and in the presence of docetaxel, bortezomib, and afatinib as unique drug classes with differing mechanisms of action. We observed persistent growth in co-cultures under drug pressure, despite previous drug sensitivity of both the WT and the mutant, demonstrating the ability for phenotypic switching in heterogenous tumors. To quantify this behavior, we developed a mathematical model of the growth dynamics in mono- and co-cultures. We assumed that previously sensitive types exhibit an increased drug tolerance in the presence of drugs through non-linear interactions. Our model successfully characterized the phenotypic switching and our results demonstrated how genotype/genotype interactions promote increased tolerance to drugs.

The empirical data presented in Fig 2 demonstrated that the mixed, heterotypic co-culture conditions resulted in improved survival of both cell lines in the mixed culture (with the exception of M3 in afatinib). Indeed, in the case of docetaxel, such behavior could be attributed to decreased cell cycling or other mechanisms that would argue against CAT as a method of thwarting drug pressure. However, the evidence for mutually beneficial growth of both cell lines, relative to vehicle control, when exposed to multiple different classes of drugs with different mechanisms of action, some of which don’t rely on cell cycling or proliferation (e.g. bortezomib), support the hypothesis of CAT and indicates that it may explain a drug-agnostic, or more universal phenomenon. While CAT is one possible explanation for the behavior observed here, there are other potential mechanisms also at play. Given these surprising empirical data, mathematical modeling of game theoretic cooperation was employed to align theoretic and empirical evidences. Indeed, the numerous instances in which the *in silico* model fit the experimental data provide support for the overall hypothesis. There is more work to be done, and understanding the mechanisms that contribute to this phenomenon still need further investigation.

Leveraging the *ex vivo* human-autologous explant platform, our results suggested that ITH affects a patient’s sensitivity to multiple anticancer drugs predominated by kinase specific as well as general cytotoxics. In the context of our findings, this means that, regardless of druggable targets, CAT may confer universal drug resistance behavior. Therefore, better-informed therapeutic interventions should be considered such as timing the sequence of drugs [9], or using combinations of rational agents based on computational modeling [53]. Indeed, many complex alternative therapeutic interventions exist, and are being tested, which could potentially address some of the challenges of CAT.

While the present study focused on theoretical models, examining the transcriptional and proteomic profile of cells in co-culture could result in novel therapeutic targets to combine with therapy and thwart CAT. Future studies might focus on growth dynamics in models of complex heterogeneity that include the microenvironment, immune cells and stromal cells. This could paint a clear picture of how CAT influences drug response. Given the evidences presented here, using strategies that can inform mutational evolution and provide single cell transcriptional profiling should be applied in parallel to a computational effort to gain a complete picture of cell-cell interactions and help guide therapeutic options for patients receiving care.

## Supporting information

S1 Supplementary information Cooperative adaptation to therapy (CAT) confers resistance in heterogeneous non-small cell lung cancer.

S1 Fig Dicer1 indel analysis. A high number of insertion and deletions (indels) were noted in the analysis of Dicer1 across the four cell lines. A significantly higher number of indels were detected in M1, M2, and M3 when compared to wild-type strains. A higher number of base insertions was detected when compared to deletions. Positions of both insertions and deletions (compared across reads) were detected. An analysis showing the ratio of insertions and deletions at the same indel site has not been performed at this time.
(TIFF)

S2 Fig NanoCulture vs. ultra low adherence (ULA) plates. a) Representative bright field images of tumor spheroids in ULA or NanoCulture plates over a 7 day culture period. b) Graph quantifies doubling rate of NSCLC cells over the course of 11 days. c) Histogram quantifies the % of cells observed growing as flat 2-D culture vs. the number of cells growing in 3-D over the course of 7 days in NanoCulture plates.
(TIF)

S3 Fig Schematic of mono- and co-culture population dynamics. In absence of drugs, monoculture dynamics were modelled as being governed by logistic growth, where the population initially grows exponentially and eventually saturates. Given the observation of a dip and rebound in the monoculture growth assays after the introduction of drugs, we constructed the monoculture model based on the assumption that phenotype/phenotype interactions could induce a ‘switch’ into a drug-tolerant subtype (modelled as intra-species competition). These phenotype/phenotype interactions were assumed to be dominated by genotype/genotype interactions (cooperative adaptation to therapy). We hypothesized that the constant fitness of each type differs in mono- and co-cultures due to differences in culture protocols and spatial constraints. Further, we assumed additional frequency-dependent cross-terms representing the interaction between the two genotypes in co-cultures. In the presence of the therapeutic stresses and other genotypes, sensitive subtypes phenotypically switch into more drug tolerant/resistant types.
(TIFF)

S4 Fig Population dynamics of 50:50 co-cultures. M1, M2, and M3 co-culture growth without drug pressure, in docetaxel, in afatinib, and bortezomib.
(TIFF)

S1 Table List of unique and shared genes from differential gene expression analysis. (XLSX)

S2 Table Parameter estimates for M1 50:50 co-cultures in absence and presence of all drugs (docetaxel, bortezomib, and afatinib. (PDF)

S3 Table Patient demographics for tumor samples used for CANscript. (PDF)

S1 File Tumor spheroid growth dynamics. Monoculture and co-culture growth data (average and standard deviations).
(XLSX)
