# Supplementary material for: Cooperative adaptation to therapy (CAT) confers resistance in heterogeneous non-small cell lung cancer
Source: PLoS Comput Biol. 2019 Aug 26;15(8):e1007278. doi: 10.1371/journal.pcbi.1007278 (PMC6709889; doi:10.1371/journal.pcbi.1007278)
Supplement: S1 Supplementary information [file pcbi.1007278.s001.pdf]

Supplementary Information: Cooperative adaptation to therapy (CAT) confers resistance in heterogeneous non-small cell lung cancer

Morgan Craig, Kamran Kaveh, Alec Woosley, Andrew S. Brown, David Goldman, Elliot Eton, Ravindra M. Mehta, Andrew Dhawan, Kazuya Arai, M. Mamunur Rahman, Sidi Chen, Martin A. Nowak, Aaron Goldman

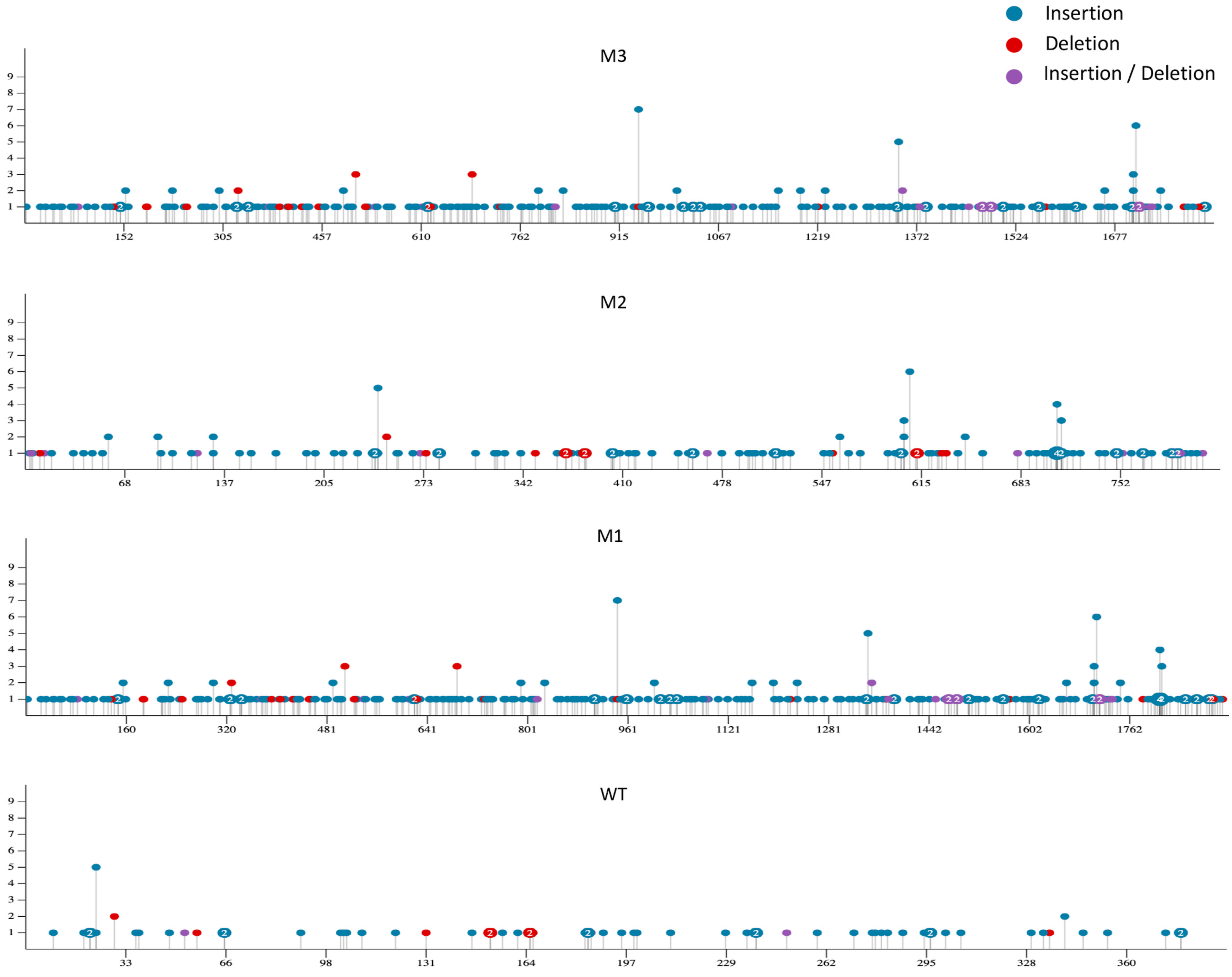

Figure S1. Dicer1 Indel Analysis. A high number of insertion and deletions (indels) were noted in the analysis of Dicer1 across the four cell lines. A significantly higher number of indels were detected in M1, M2, and M3 when compared to wild-type strains. A higher number of base insertions was detected when compared to deletions. Positions of both insertions and deletions (compared across reads) were detected. An analysis showing the ratio of insertions and deletions at the same indel site has not been performed at this time.

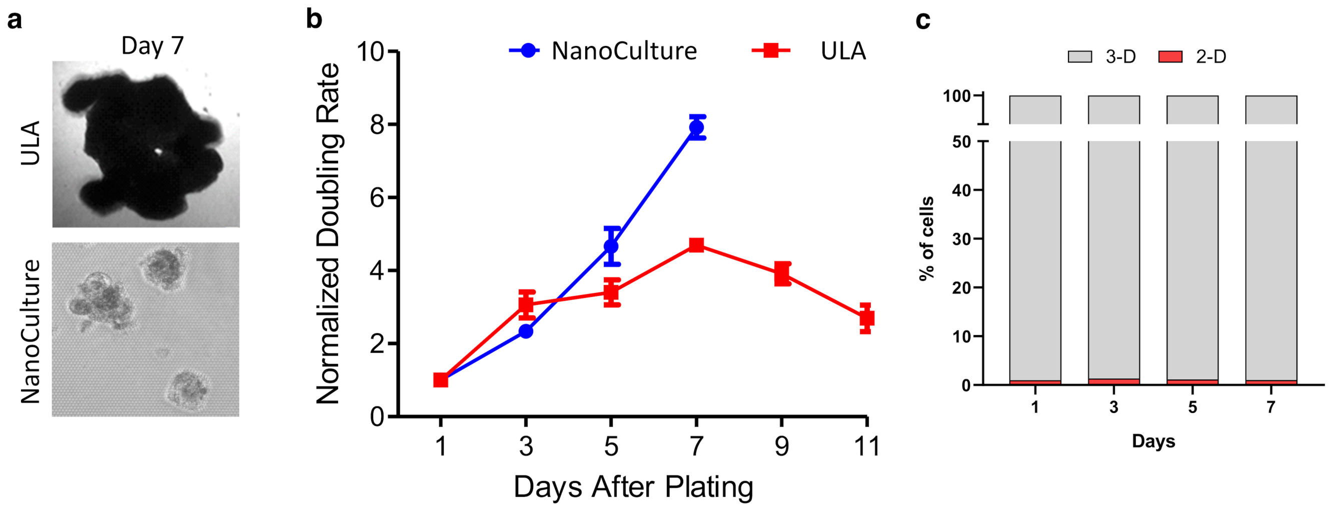

Figure S2. NanoCulture vs. ultra low adherence (ULA) plates. a) Representative bright field images of tumor spheroids in ULA or NanoCulture plates over a 7 day culture period. b) Graph quantifies doubling rate of NSCLC cells over the course of 11 days. c) Histogram quantifies the % of cells observed growing as flat 2-D culture vs. the number of cells growing in 3-D over the course of 7 days in NanoCulture plates.

General modelling framework

We begin with a homogenous model where cells divide with rate b and die with a constant rate d. The death rate d is partly related to the apoptosis rate of individual cells and has a component that is strongly dependent on microenvironmental conditions. At higher frequencies/populations, the reproduction rate is reduced and kept constant at the carrying capacity $\tilde{K}$. During treatment, cytotoxic chemo-drugs significantly increase the value of d, with its exact value depending on the dose, the drug-specific dose-response, as well as other microenvironmental parameters (including nutrient availability). The ordinary differential equation that describes such dynamics is the usual logistic equation written as

$$\frac{\mathrm{d}n}{\mathrm{d}t} = bn\left(1 - \frac{n}{\tilde{K}}\right) - dn. \tag{S1}$$

This model can be re-parametrized in terms of a linear fitness term defined as the effective rate of birth minus death $(b - d)$ and a new carrying capacity value K

$$\begin{aligned}\frac{\mathrm{d}n}{\mathrm{d}t} &= (b-d)n - \frac{b}{\tilde{K}}n^2 \\ &= (b-d)n\left(1 - \frac{1}{K}n\right),\end{aligned}$$

where the new carrying capacity K is defined as $K = \tilde{K}(1-d/b)$. If the death rate is larger than the birth rate, fitness decreases, i.e. the overall growth rate is negative. The value of effective carrying capacity K is also changed (however, the quantitative change in the value of K does not have a direct consequence within the framework of our model). The value $b - d$ can then be reinterpreted as a (linear) fitness or growth rate l.

Consider now heterogeneous growth dynamics where more than one genotype grows, and types can interact with one another. We denote parameters for each type with a subscript i. Cross-terms, representing genotype-genotype interactions, further contribute to the quadratic terms above as

$$\frac{\mathrm{d}n_i}{\mathrm{d}t} = b_i n_i\left(1 - \frac{n_i}{\tilde{K}}\right) - d_i n_i + \tilde{m}_{ij} n_i n_j,$$

where i =(WT,Mut). Upon re-parameterization, we obtain the cross-term coefficients (payoffs) as well as carrying capacity:

$$\begin{aligned}\frac{\mathrm{d}n_i}{\mathrm{d}t} &= (b_i - d_i)n_i - \frac{b}{\tilde{K}}n_i^2 + \tilde{m}_{ij} n_i n_j \\ &= (b_i - d_i)n_i\left(1 - \frac{1}{K}n_i + a_{ij}n_j\right).\end{aligned} \tag{S2}$$

Similar to the effective carrying capacity, the new payoff value (a_{ij}) is defined as $\tilde{m}_{ij} = a_{ij}(b_i - d_i)$. Since we did not assume that payoff values are symmetric, (i.e. $a_{ij} \neq a_{ji}$), the new parametrization in terms of $l_i = b_i - d_i$, K_i, and a_{ij} is general and equivalent to the model in Eq. (S1) (or Eq. (S2) for two genotypes).

CAT model of adaptive resistance

Each genotype (WT or Dicer1 mutant) was considered to have two phenotypically distinct variants: a sensitive type that is abundant in the absence of the treatment and a drug tolerant type. In the presence of environmental stresses (like chemotherapeutic drugs), sensitive cells can phenotypically switch and become

drug tolerant. To model the differences in population dynamics in mono- and co-culture we adapted the evolutionary game theoretic framework of replicator dynamics, where the game interaction payoffs describe the fitness gain or loss of sensitive/tolerant cell phenotypes in the presence of phenotype/phenotype or genotype/genotype interactions. Let x_{WT} be the number of wild-type cells and x_{M_i} be the number of mutant cells of type i ($i = 1, 2$). Assuming that WT and mutants initially grow exponentially to eventual saturations and that the dynamics in the presence of drugs are regulated by phenotypic transitions to a tolerant subtype, the monoculture model is described by

$$\begin{aligned}\frac{dx_{type}^{sens}}{dt} &= l_{type}^{sens} x_{type}^{sens}\left(1 - \frac{(x_{type}^{sens} + x_{type}^{tol})}{K_{type}}\right) - \nu_{type} x_{type}^{sens}\\ \frac{dx_{type}^{tol}}{dt} &= l_{type}^{tol} x_{type}^{tol}\left(1 - \frac{(x_{type}^{sens} + x_{type}^{tol})}{K_{type}}\right) + \nu_{type} x_{type}^{sens}\end{aligned} \tag{S3}$$

where the superscript *sens* describes the drug-sensitive phenotype, and *tol* the drug-tolerant phenotype, K_{type} denotes the carrying capacity, and ν_{type} measures the intra-species switching rate. Note that no backwards switching from tolerant to sensitive is considered.

To model co-cultures between WT and either M1, M2, or M3 mutant cells (in 10:90, 50:50, and 90:10 WT:mutant proportions), we assumed that intra-species interactions are dominated by inter-species relationships, that is that the adaptive behaviors of cells in co-culture are dictated by CAT dynamics. Accordingly, as in the monoculture case, let l_1 and l_2 be the constant fitnesses, and $K_{WT_{co}}$ and $K_{Mi_{co}}$ be the carrying capacities of the wild-type and mutant populations, respectively. We modified Eq. (S3) by assuming the growth rates of each population are adjusted through their interactions with the other via the cross-terms a_{12} and a_{21} that represent the interactions between wild-type and mutants, respectively. Note that we do not restrict the model to be fully competitive *a priori*, so $a_{12}, a_{21} \in \mathbb{R}$. The co-culture dynamics are then given as

$$\begin{aligned}\frac{dx_{\mathrm{WT}}}{dt} &= l_1 x_{\mathrm{WT}}\left(1 - \frac{x_{\mathrm{WT}}}{K_{WT_{co}}} + a_{12} x_{\mathrm{M}_i}\right)\\ \frac{dx_{\mathrm{M}_i}}{dt} &= l_2 x_{\mathrm{M}_i}\left(1 - \frac{x_{\mathrm{M}_i}}{K_{Mi_{co}}} + a_{21} x_{\mathrm{WT}}\right)\end{aligned} \tag{S4}$$

The mono- and co-culture model structures are schematically represented in Figure S3.

Parameter selection and optimization

For monocultures modelled by Eq. (S3) in the absence and the presence of drugs, we began by calculating the average of each population per cell type and per drug, if applicable. The Matlab [1] function *fmincon* was used to minimize the cost function c given by conventional least squares regression:

$$c = \frac{1}{n}\sum_{j=1}^{n}(y_j - \hat{y}_j)^2 \tag{S5}$$

where y_j is a vector of predicted values, $\hat{y}_j$ is a vector of observed values, and n is the total number of data points. We employed a global search algorithm to estimate parameters using the *MultiStart* optimization routine in Matlab [1]. 100 initial points were generated and each were optimized using the *fmincon* solving routine with error measured by Eq. S5. The optimal set of parameters was then selected by minimizing the resulting cost function values from each of the 100 runs. This methodology was selected to reduce the influence of initial conditions on the outcome, given the rugged parameter landscape.

To estimate parameters from the co-culture experiments, we employed a similar least-squares cost

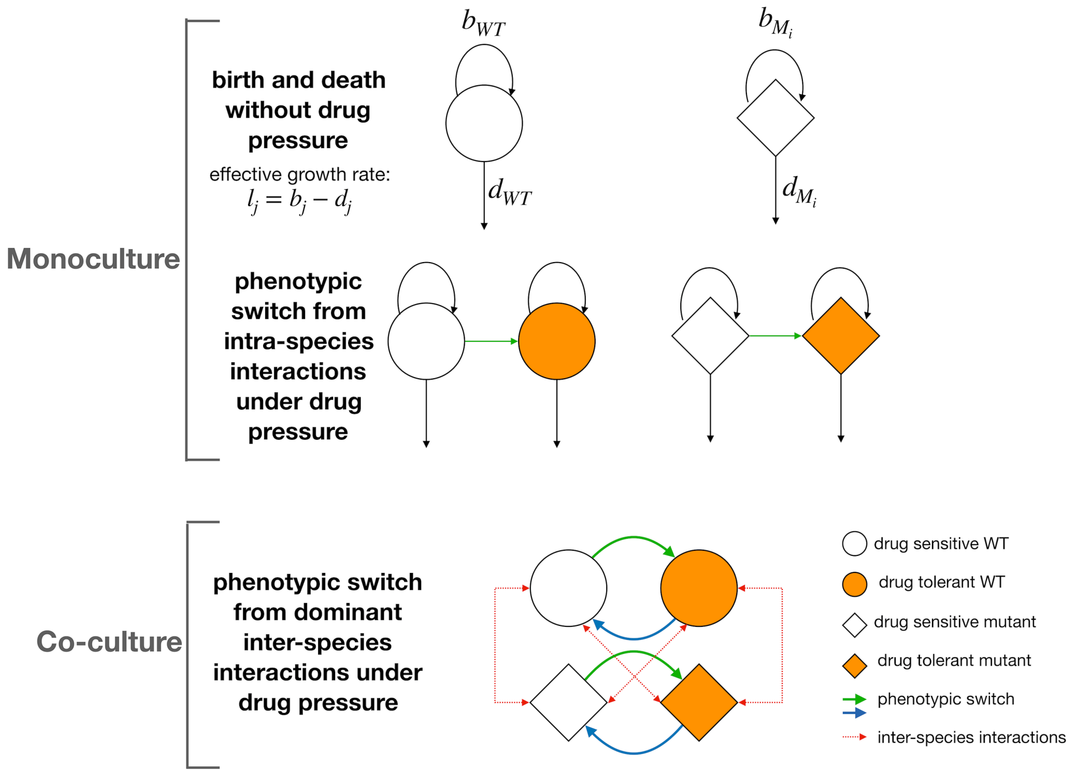

Figure S3. Schematic of mono- and co-culture population dynamics In absence of drugs, monoculture dynamics were modelled as being governed by logistic growth, where the population initially grows exponentially and eventually saturates. Given the observation of a dip and rebound in the monoculture growth assays after the introduction of drugs, we assumed that phenotype/phenotype interactions could induce a switch into a drug-tolerant subtype (modelled as intra-species competition). These phenotype/phenotype interactions were assumed to be dominated by genotype/genotype interactions (cooperative adaptation to therapy). We hypothesized that the constant fitness of each type differs in mono- and co-cultures due to differences in culture protocols and spatial constraints. Further, we assumed additional frequency-dependent cross-terms representing the interaction between the two genotypes in co-cultures. In the presence of the therapeutic stresses and other genotypes, sensitive subtypes phenotypically switch into more drug tolerant/resistant types.

function as in Eq. (S5)

$$c = \frac{1}{2n} \sum_{j=1}^{n} \left(\left(x_{\mathrm{WT}_j} - \hat{x}_{\mathrm{WT}_j} \right)^2 + \left(x_{\mathrm{M}_{i\,j}} - \hat{x}_{\mathrm{M}_{i\,j}} \right)^2 \right), \tag{S6}$$

where x_{WT_j} is the predicted number and $\hat{x}_{\mathrm{WT}_j}$ is the observed number of wild-type cells at time j. Similarly, $x_{\mathrm{M}_{i\,j}}$ is the predicted and $\hat{x}_{\mathrm{M}_{i\,j}}$ is the observed number of mutant i type cells at time j. As in the monoculture case, Eq. (S6) was applied in 100 *fmincon* calls within the *MultiStart* global optimization routine. The resulting cost functions were minimized to find the optimal set of parameters in each co-culture case. Parameter identifiability can be a concern in systems of linear, ordinary differential equations with fewer observations than equations. Here we have information for both the WT and mutants in each experiment but, as noted above, the parameter landscape is rugged and it can be difficult to find a global optimal set of parameters. To reduce concerns about structural identifiability, in addition to using the *MultiStart* optimization routine to lessen the influence of the parameter landscape, we performed pairwise fits to WT and a single mutant at a time. We further employed a step-wise approach to parameter

estimation, summarized below. Representative fitting results for 50:50 co-cultures across all mutants without and with drugs are provided in S4 Fig.

1. **Estimate monoculture parameters l_{type}^{sens} and K_{type}^{sens} (Eq. (S3)) from no drug growth assays**
2. **Estimate monoculture parameters l_{type}^{sens}, l_{type}^{tol} and ν_{type} (Eq. (S3)) from growth assays exposed to drugs (K_{type}^{sens} remain fixed from no drug case)**
3. **For each initial proportion of cells (10:90, 50:50, and 90:10 WT:mutuant), estimate co-culture parameters l_1, l_2, and K_{type} from Eq. (S4):**
   (a) for x_{WT}, $x_{\mathrm{M_1}}$, and $x_{\mathrm{M_2}}$ in the absence of drugs
   (b) for x_{WT}, $x_{\mathrm{M_1}}$, and $x_{\mathrm{M_2}}$ in the presence of docetaxel, bortezomib, and afatinib

An assumption about the shifting dynamics between mono- and co-cultures, without and with drugs, should be noted. We observed marked differences in growth between monocultures and co-cultures of wild-types and each mutant in the absence and presence of drugs, regardless of initial proportions. This led us to believe that the introduction of a second cell-type inhibits growth due to environmental constraints (lack of space etc.). To test this hypothesis, we refit the constant growth rates l_i and added the cross-interaction terms a_{ij} $(i, j = 1, 2)$ from the monoculture to co-culture case. As indicated in S2 Table, this impression was borne out. We further posited that adaptive resistance phenotypes were emerging when co-cultures were exposed to any of the chemotherapeutic drugs. Therefore, beyond adjustments in constant growth rates in each population from the no-drug case, cross-competition terms would be altered in drug co-cultures. Thus all parameters in Eq. (S4) were re-estimated for every individual experiment, allowing us to draw conclusions about how dynamics changed with changing experimental conditions.

**Parameter**	**No Drug**	**Docetaxel**	**Bortezomib**	**Afatinib**
l_1	0.4756	0.4756	0.0699	0.5487
l_2	0.3694	0.3964	0.0058	0.0132
K_{WT}	47.62	250	14.35	1,000
K_{M_2}	250	1E6	452,693.53	28.82
a_{12}	-	0.0015	0.2	-0.0007
a_{21}	-	-0.0004	0.0285	-0.0049
Error	0.7891	0.0031	1.519	2.1777×10^{-7}

Table S2. Parameter estimates for M1 50:50 co-cultures in absence of and presence all drugs (docetaxel, bortezomib, and afatinib).

References

1. Mathworks. MATLAB 2018a; 2018.

Patient ID	Cancer Type	Cancer Stage	Anatomic Site of Specimen
MB-077	Non-Small Cell Lung Cancer (NSCLC)	Metastatic	Liver
MB-060	Non-Small Cell Lung Cancer (NSCLC)	Metastatic	Lung
MB-103	Non-Small Cell Lung Cancer (NSCLC)	Metastatic	Lung
MB-104	Non-Small Cell Lung Cancer (NSCLC)	Stage IV	Lung
MB-048	Non-Small Cell Lung Cancer (NSCLC)	Stage IV	Lung
MB-102	Non-Small Cell Lung Cancer (NSCLC)	Metastatic	Right abdominal wall nodule
MB-101	Non-Small Cell Lung Cancer (NSCLC)	Metastatic	Left abdominal wall nodule
MB-003	Non-Small Cell Lung Cancer (NSCLC)	Stage III	Lung

Table S3. Patient demographics for tumor samples used for CANscript

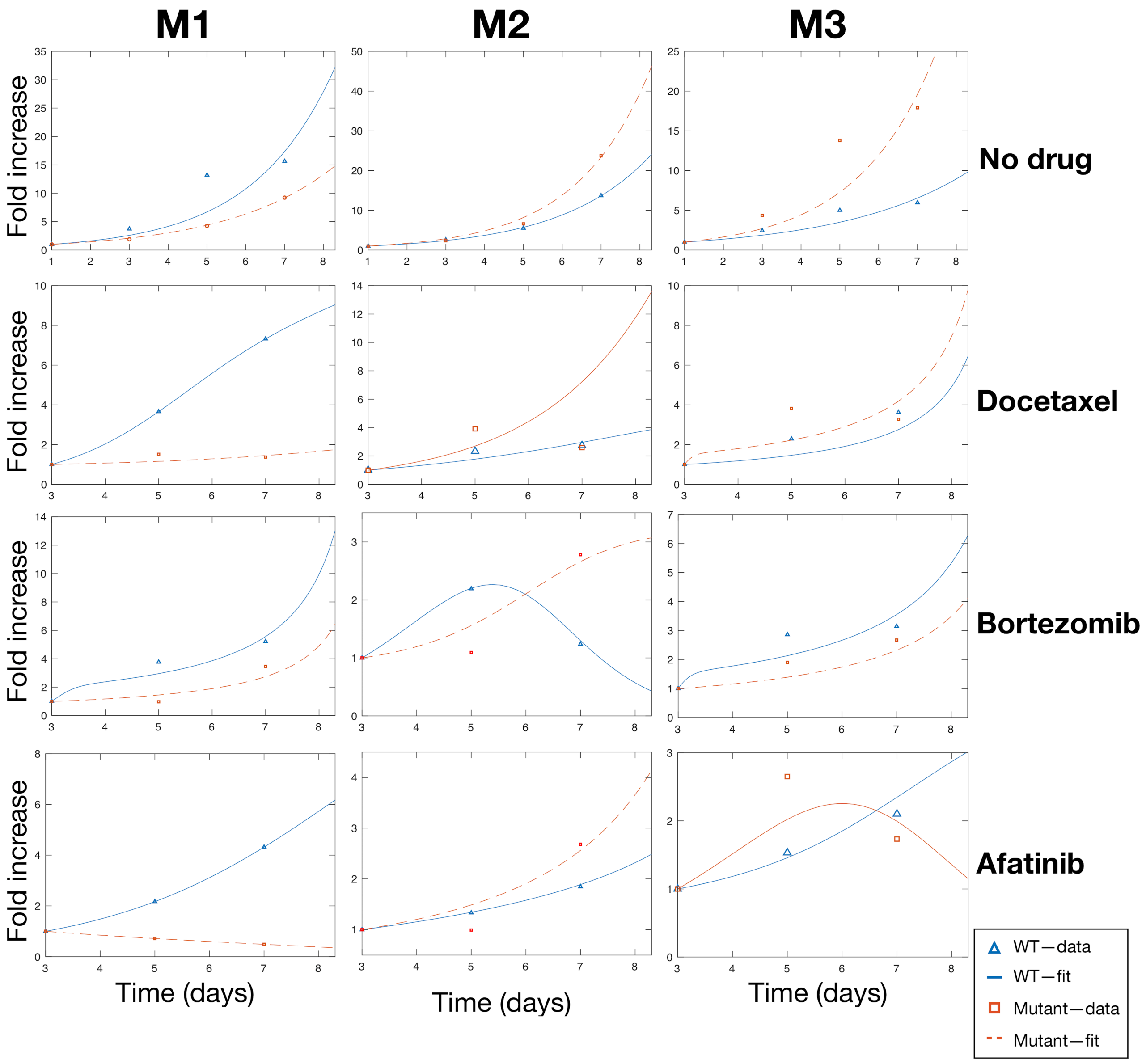

Figure S4. Population dynamics of 50:50 co-cultures M1, M2, and M3 co-culture growth without drug pressure, in docetaxel, in afatinib, and bortezomib.
