# Supplementary figures and images for: Cooperative adaptation to therapy (CAT) confers resistance in heterogeneous non-small cell lung cancer
Source: PLoS Comput Biol. 2019 Aug 26;15(8):e1007278. doi: 10.1371/journal.pcbi.1007278 (PMC6709889; doi:10.1371/journal.pcbi.1007278)

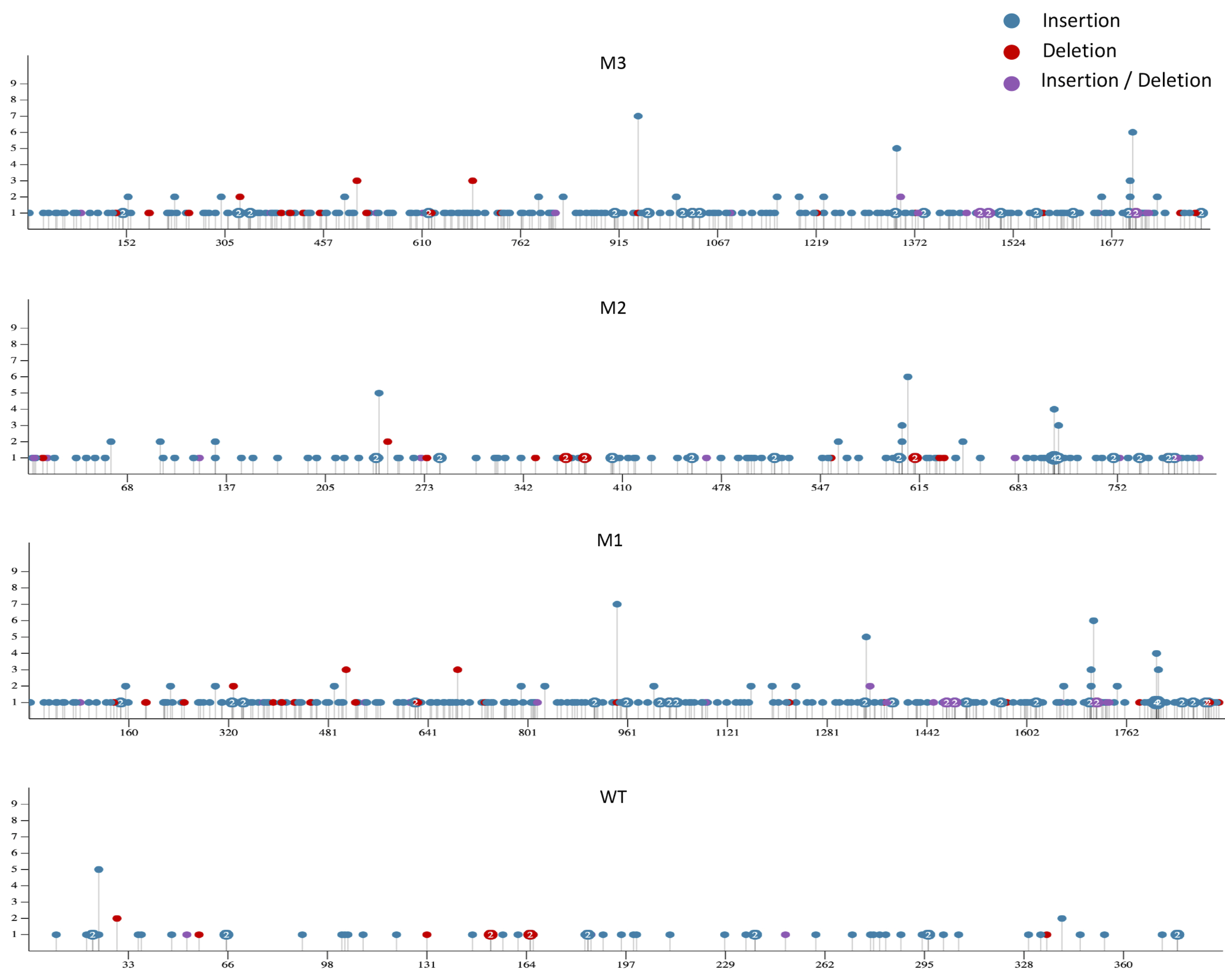

Supplement: S1 Fig — A high number of insertion and deletions (indels) were noted in the analysis of Dicer1 across the four cell lines. A significantly higher number of indels were detected in M1, M2, and M3 when compared to wild-type strains. A higher number of base insertions was detected when compared to deletions. Positions of both insertions and deletions (compared across reads) were detected. An analysis showing the ratio of insertions and deletions at the same indel site has not been performed at this time. (TIFF) [file pcbi.1007278.s002.tiff]

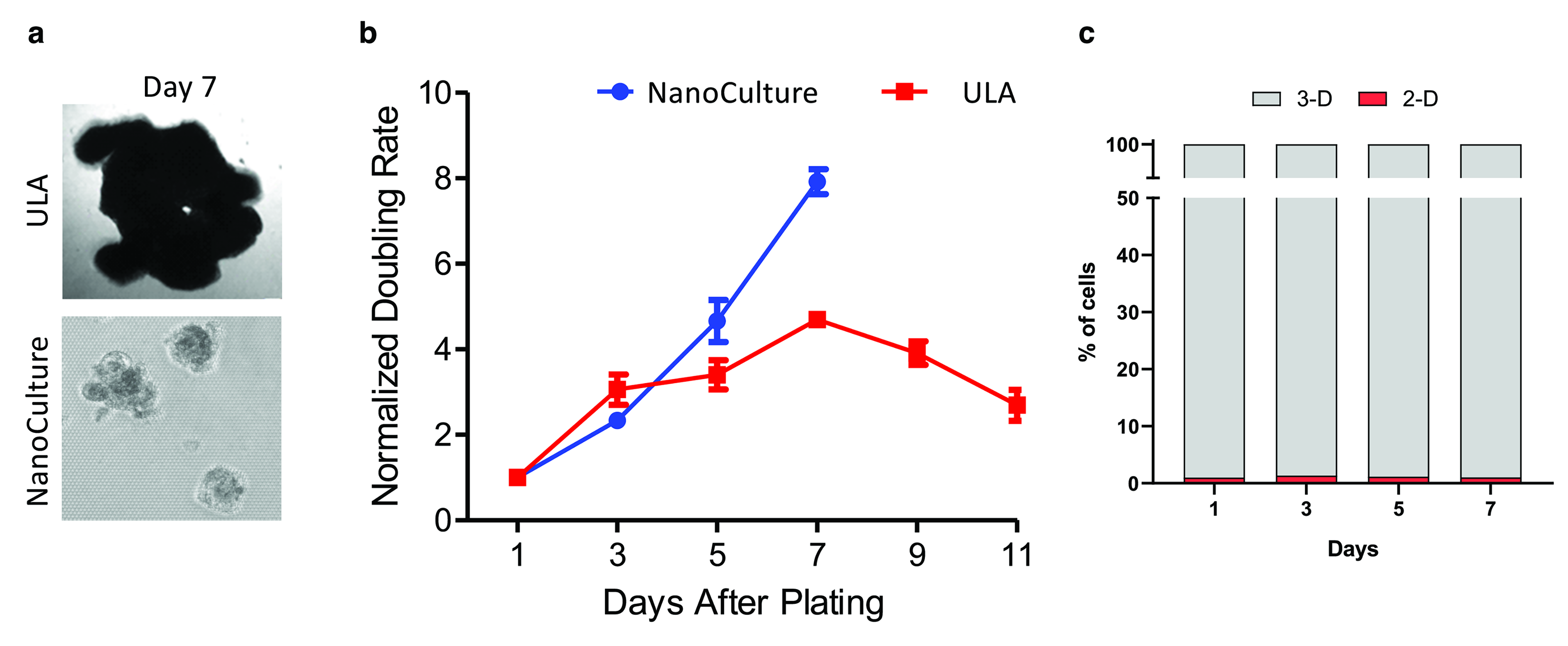

Supplement: S2 Fig — a) Representative bright field images of tumor spheroids in ULA or NanoCulture plates over a 7 day culture period. b) Graph quantifies doubling rate of NSCLC cells over the course of 11 days. c) Histogram quantifies the % of cells observed growing as flat 2-D culture vs. the number of cells growing in 3-D over the course of 7 days in NanoCulture plates. (TIF) [file pcbi.1007278.s003.tif]

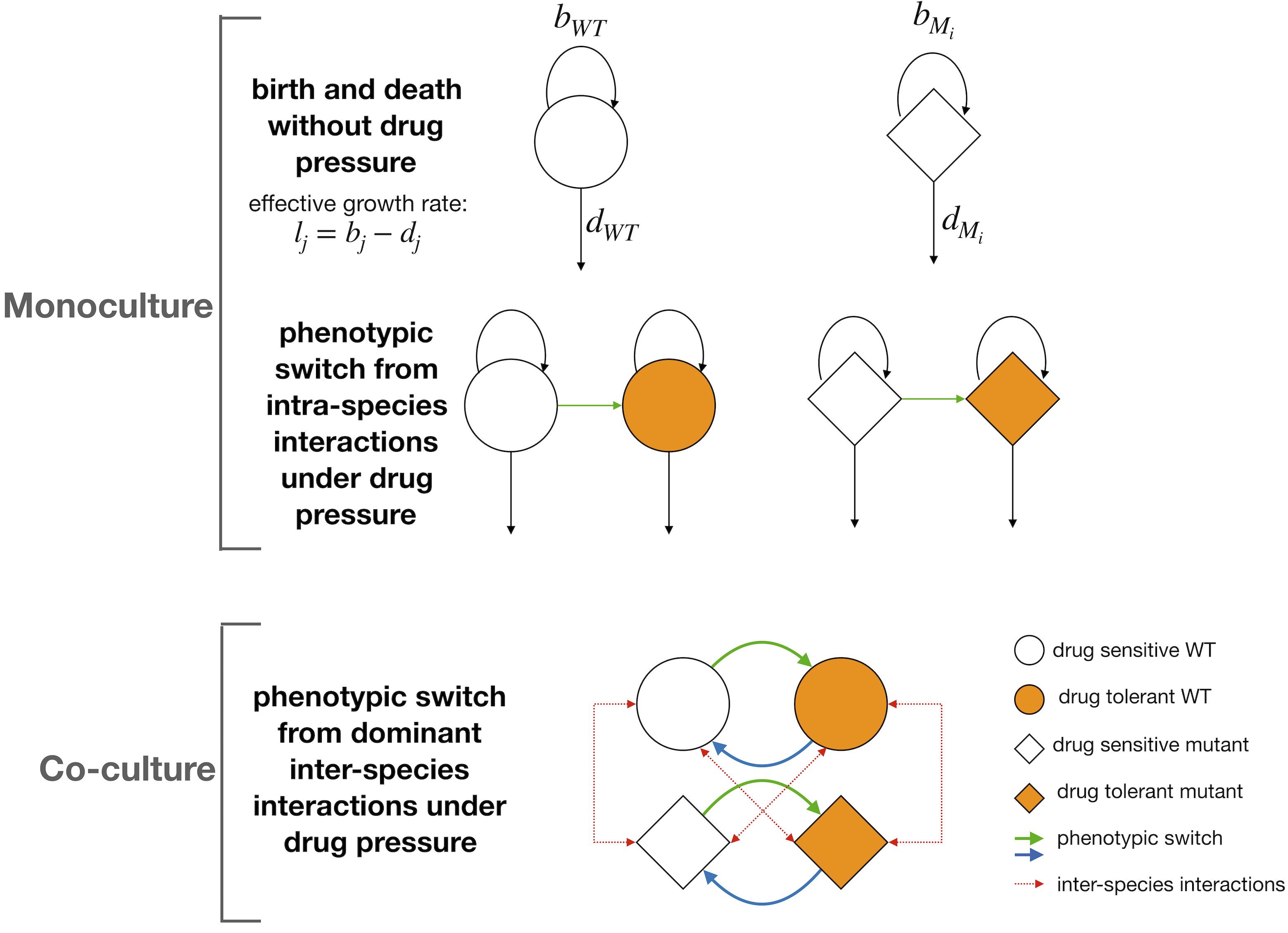

Supplement: S3 Fig — In absence of drugs, monoculture dynamics were modelled as being governed by logistic growth, where the population initially grows exponentially and eventually saturates. Given the observation of a dip and rebound in the monoculture growth assays after the introduction of drugs, we constructed the monoculture model based on the assumption that phenotype/phenotype interactions could induce a ‘switch’ into a drug-tolerant subtype (modelled as intra-species competition). These phenotype/phenotype interactions were assumed to be dominated by genotype/genotype interactions (cooperative adaptation to therapy). We hypothesized that the constant fitness of each type differs in mono- and co-cultures due to differences in culture protocols and spatial constraints. Further, we assumed additional frequency-dependent cross-terms representing the interaction between the two genotypes in co-cultures. In the presence of the therapeutic stresses and other genotypes, sensitive subtypes phenotypically switch into more drug tolerant/resistant types. (TIFF) [file pcbi.1007278.s004.tiff]

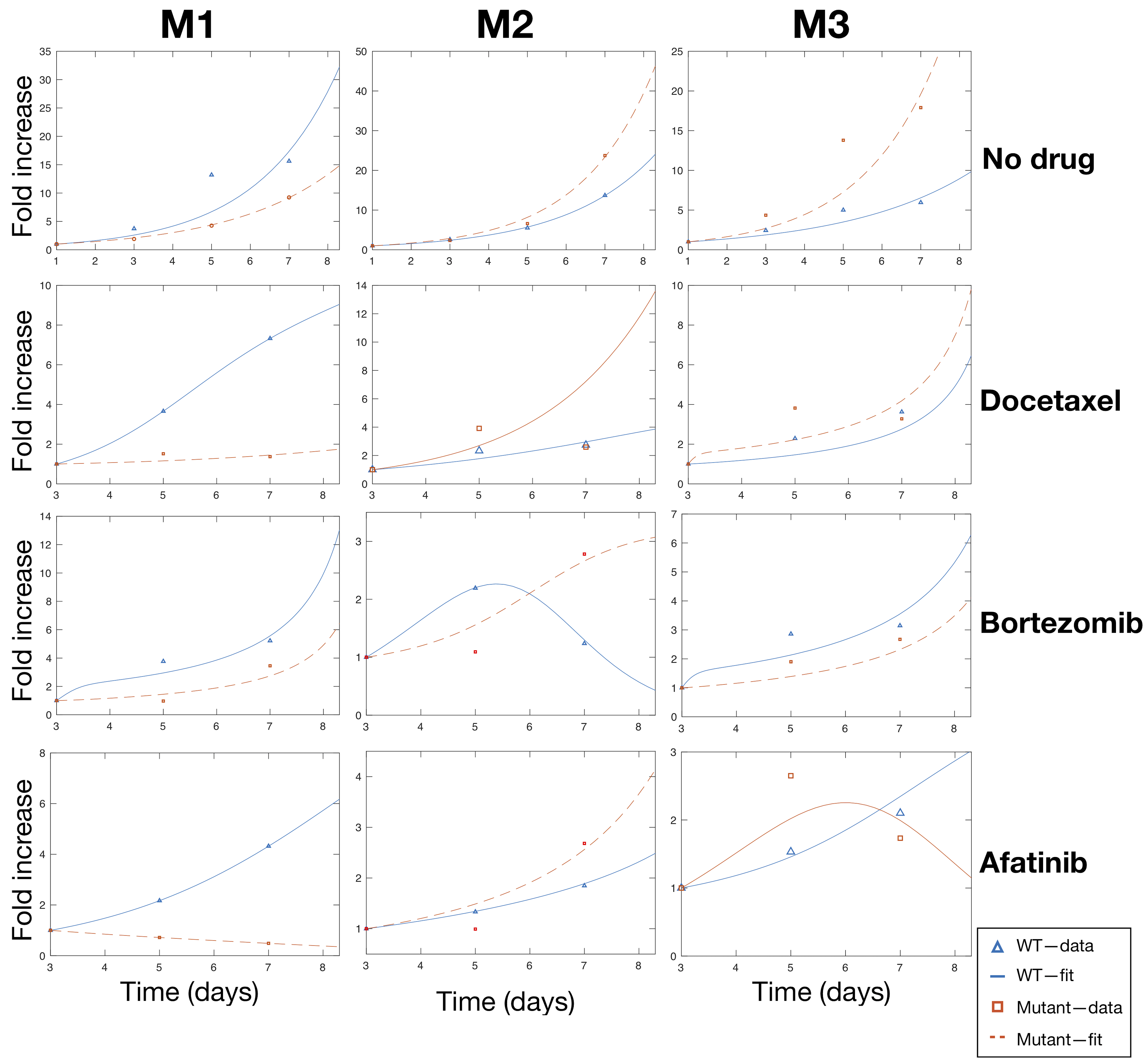

Supplement: S4 Fig — M1, M2, and M3 co-culture growth without drug pressure, in docetaxel, in afatinib, and bortezomib. (TIFF) [file pcbi.1007278.s005.tiff]
