# Supplementary material for: Cooperative adaptation to therapy (CAT) confers resistance in heterogeneous non-small cell lung cancer
Source: PLoS Comput Biol. 2019 Aug 26;15(8):e1007278. doi: 10.1371/journal.pcbi.1007278 (PMC6709889; doi:10.1371/journal.pcbi.1007278)
Supplement: S2 Table — (PDF) [file pcbi.1007278.s007.pdf]

Parameter	No Drug	Docetaxel	Bortezomib	Afatinib
l_1	0.4756	0.4756	0.0699	0.5487
l_2	0.3694	0.3964	0.0058	0.0132
K_{WT}	47.62	250	14.35	1,000
K_{M_2}	250	1E6	452,693.53	28.82
a_{12}	-	0.0015	0.2	-0.0007
a_{21}	-	-0.0004	0.0285	-0.0049
Error	0.7891	0.0031	1.519	2.1777×10^{-7}
