# Supplementary material for: Cooperative adaptation to therapy (CAT) confers resistance in heterogeneous non-small cell lung cancer
Source: PLoS Comput Biol. 2019 Aug 26;15(8):e1007278. doi: 10.1371/journal.pcbi.1007278 (PMC6709889; doi:10.1371/journal.pcbi.1007278)
Supplement: S3 Table — (PDF) [file pcbi.1007278.s008.pdf]

Patient ID	Cancer Type	Cancer Stage	Anatomic Site of Specimen
MB-077	Non-Small Cell Lung Cancer (NSCLC)	Metastatic	Liver
MB-060	Non-Small Cell Lung Cancer (NSCLC)	Metastatic	Lung
MB-103	Non-Small Cell Lung Cancer (NSCLC)	Metastatic	Lung
MB-104	Non-Small Cell Lung Cancer (NSCLC)	Stage IV	Lung
MB-048	Non-Small Cell Lung Cancer (NSCLC)	Stage IV	Lung
MB-102	Non-Small Cell Lung Cancer (NSCLC)	Metastatic	Right abdominal wall nodule
MB-101	Non-Small Cell Lung Cancer (NSCLC)	Metastatic	Left abdominal wall nodule
MB-003	Non-Small Cell Lung Cancer (NSCLC)	Stage III	Lung
